# Biodegradable Microplastics from Agricultural Mulch Films: Implications for Plant Growth-Promoting Bacteria and Plant’s Oxidative Stress

**DOI:** 10.3390/antiox14020230

**Published:** 2025-02-18

**Authors:** Bruno Carneiro, Paula Marques, Tiago Lopes, Etelvina Figueira

**Affiliations:** 1 Department of Biology, University of Aveiro, 3810-193 Aveiro, Portugal; brunomcarneiro@ua.pt; 2 TEMA—Centre for Mechanical Technology and Automation, Department of Mechanical Engineering, University of Aveiro, 3810-193 Aveiro, Portugal; paulam@ua.pt; 3 CESAM—Centre for Environmental and Marine Studies, Department of Biology, University of Aveiro, 3810-193 Aveiro, Portugal; tslopes@ua.pt

**Keywords:** biodegradable microplastics, PGPB, agriculture, environment, oxidative stress, antioxidant enzymes

## Abstract

This study explores the interactions between biodegradable (BIO) microplastics and plant growth-promoting bacteria (PGPB), assessing their effects on soil health and crop productivity. Five bacterial strains, *Bacillus*, *Enterobacter*, *Kosakonia*, *Rhizobium*, and *Pseudomonas*, were exposed to BIO microplastics to examine strain-specific responses. This study revealed that while most bacteria experienced growth inhibition, *Kosakonia* sp. O21 was poorly affected by BIO microplastics, indicating a potential for microplastic degradation. This study further investigated the effect of these microplastics on plant growth and biochemistry. Results showed that exposure to BIO microplastics significatively reduced plant growth and caused oxidative stress, affecting membranes and proteins and inducing the activity of glutathione S-transferases (GSTs), catalase (CAT), and superoxide dismutase (SOD) as antioxidant responses. Bacterial inoculation alleviated plant oxidative stress, especially at lower concentrations of microplastics. These findings emphasize the critical role of oxidative stress in mediating the negative effects of BIO microplastics on plants and the relevance of bacterial strains that can tolerate BIO microplastics to protect plants from BIO microplastics’ effects. Results also highlight the importance of extending research to assess the long-term implications of biodegradable microplastics for soil PGPBs and plant health and crop productivity. This study contributes to sustainable agricultural practices by offering insights into mitigating the risks of microplastic pollution through microbial-based interventions.

## 1. Introduction

Biodegradable plastic mulch (BDM) films have recently gained much attention due to the development of sustainable alternatives to conventional polyethylene (PE) mulch in agricultural systems. These films offer considerable advantages in terms of a reduction in plastic waste accumulation within the environment while retaining many of the beneficial properties of conventional plastic mulches, namely improvement in soil temperature, suppression of weeds, and conservation of soil moisture [1]. What makes them unique is that they are meant to completely degrade into natural components such as water, carbon dioxide, and biomass while in the soil [2]. This property represents a promise to solve one of the major environmental problems that ecosystems are currently facing: plastics persistence. However, the full biodegradability of biodegradable plastics and their true impacts on soil health remain active areas of research and debate, since the degradation of BDM films is based on several factors, such as material composition, environmental conditions, and microbial activity in the soil [3]. Usually made from PLA (polylactic acid), PHA (polyhydroxyalkanoates), or starch-based compounds, BDMs are usually designed to break down more readily than conventional plastics [4]. Yet, there is growing concern that incomplete degradation of these materials might still result in the formation of microplastics or other residues that remain in the soil. Even biodegradable polymers may pose risks when released into agricultural soils, as their degradation products may affect soil structure, microbial communities, and plant growth in ways that are not fully understood [5,6]. Biodegradable plastic mulch films also have their specific problems. The rates of degradation are highly dependent on the local conditions of soil type, moisture, temperature, and specific microorganisms present in the environment that have the capability to degrade the material [3]. Therefore, BDMs may take a lot more time to break down and could accumulate fragments over time, with possible long-term negative effects on soil quality. The potential risks mentioned above underline the need for further in-depth research on the environmental behaviour of BDMs, the development of new testing methodologies to assess their biodegradability under realistic agricultural conditions, and their effects on plant growth-promoting bacteria (PGPB), which represent a promising tool for a more sustainable agriculture.

Soils are the basis of agricultural productivity; therefore, it is important that the influence of BDMs on key soil processes like nutrient cycling, water retention, and microbial activity be elucidated when underpinning the potential for BDMs as a viable option for sustainable agriculture. A deeper knowledge of the complete life cycle of BDM films from manufacturing to degradation allows agricultural systems to benefit from this technology and its potential use in conjunction with other ecofriendly technologies, while it reduces environmental risks associated with the use of plastics. For that reason, the characterization of BDMs and an extensive study of their effects on microbial agricultural inoculants is of paramount importance for healthy soils and a sustainable agriculture. Therefore, in order to comprehend BDMs’ effects on plant growth, this study aims to evaluate the interactions between biodegradable microplastics and plant growth-promoting bacteria. This research also intends to identify bacteria that can tolerate and/or degrade BIO (biodegradable) microplastics and that can lessen their detrimental effects on plant growth and biochemical status. To reduce the impact that BIO microplastic contamination may impose on crops and advance sustainable farming methods, this study seeks to shed light on microbial-based approaches.

## 2. Material and Methods

### 2.1. Production of BIO Particles

Full characterization of biodegradable microplastics and their effects on PGPB is a very important aspect in the advancement of our understanding of the role played by microplastics in soil health and agricultural productivity.

#### 2.1.1. Materials

The type of biodegradable plastic mulch used for the purpose of this thesis was Agrobiofilm^®^, a plastic film with Mater-Bi^®^ (Novamont, Novara, Italy) formulation, colour black, and 12 μm thick, that was graciously supplied by the company Silvex^®^ (Benavente, Portugal), being certified as biodegradable and compostable (NF U52-001, 2005; Agrobiofilm, 2013).

#### 2.1.2. Methodology

In this study, the plastic mulch film underwent separation into specimens subjected to weathering with ultraviolet (UV) radiation exposure and specimens not exposed. The specimens designated for non-exposure were promptly processed to undergo conversion into microplastics, advancing to the milling phase. During this phase, BIO mulch film was initially fragmented into smaller segments using a blade. Subsequently, the resulting fragments were further comminuted using a hand blender Bosch MSM2610B (Bosch, Gerlingen, Germany) for 30 s. Subsequent size reduction was achieved through successive cycles of 30 s blending, followed by grinding in a coffee grinder Tristar KM-2270 (Tristar, Tilburg, The Netherlands). To separate the mixture of micro and macroplastics, the milled samples underwent sieving using a series of sieves with mesh sizes measuring 2.8 mm, 2 mm, 1 mm, 500 µm, and 250 µm. Ten grams of the material were introduced into the upper compartment of the sieving apparatus and subjected to vibration at an amplitude of 50 Hz for a duration of 1 h and 20 min, given the exceedingly light weight of the material. Subsequently, the separated fractions were weighed to ascertain the distribution of each size interval microplastics.

For plastics designated for weathering, an additional initial step was implemented wherein BIO plastic mulch film underwent in a Q-Lab QUV (Q-Lab, Westlake, OH, USA) accelerated weathering tester, a 500-h cycle of UVA radiation at 340 nm, operating at an intensity of 0.77 W/m^2^. This exposure regimen comprised alternating cycles of 4 h of UV exposure followed by 4 h without UV exposure but with condensation occurring at 50 °C, conforming to the guidelines outlined in ISO 4892 [7] for assessing plastic UV weathering.

#### 2.1.3. Analysis and Characterization of Mulch Particles

The BIO microplastic particles generated were subjected to scanning electron microscope (SEM) analysis and energy dispersive spectrometry (EDS) analysis (Hitachi TM4000Plus II, Hitachi, Japan). Subsequently, micro-Fourier Transform Interferometer (μ-FTIR) analysis was employed to identify the composition of the microplastics using attenuated total reflectance (ATR). The μ-FTIR spectrometer (PerkinElmer’s Spectrum Two™ IR spectrometer, PerkinElmer, Shelton, CT, USA) spectra were recorded from wavenumbers ranging from 400 to 4000 cm^−1^. The TG analysis was carried out using a NETZSCH Simultaneous Thermal Analyzer 449F3 (NETZSCH, Selb, Germany). The TG experiments were performed in ambient air on an open Al_2_O_3_ crucible at a heating rate of 10 °C/min and a temperature range of 30–800 °C. The reference container was an empty Al_2_O_3_ crucible [8] The surface charge of BIO microparticles was measured by a zeta potential tester (Zetasizer Nano-ZS 90, Malvern Instruments Ltd., Malvern, Worcestershire, UK), as described by [9].

### 2.2. Bacterial Strains, Growth Conditions, and Tolerance

#### 2.2.1. Bacterial Strains

*Bacillus* sp. strain J25 was previously isolated from the root nodules of *Phaseolus vulgaris* L. The 16S rRNA gene was amplified and the PCR products sequenced and used to identify the bacterium strain to genus level. The partial 16S rRNA gene sequence was deposited in GenBank (Accession: MK713663.1). *Bacillus* sp. strain J25 was previously described as having high proteolytic, amylolytic, and lipolytic capacities [10]. The strain was grown overnight at 26 °C in an orbital shaker (160 rpm) in tubes containing 5 mL of Yeast Mannitol Broth (YMB) medium with sucrose as a carbon source instead of mannitol [11].

*Enterobacter ludwigii* sp. strain C11 was previously isolated from the root nodules of *P. vulgaris* L. The 16S rRNA gene was amplified and the PCR products sequenced and used to identify the bacterium strain to genus level, being more recently identified to the species level. The partial 16S rRNA gene sequence was deposited in GenBank (Accession: MK318777.1). *E. ludwigii* species was previously described as potentially able to degrade PE [12] and, according to our laboratory team, to have high ability to solubilize phosphate [13]. The strain was grown in tubes containing 5 mL of Yeast Mannitol Broth (YMB) medium overnight at 26 °C in an orbital shaker (160 rpm) [11].

*Kosakonia* sp. O21 strain was previously isolated from the root nodules of *P. vulgaris* L. The 16S rRNA gene was amplified and the PCR products sequenced and used to identify the bacterium strain to genus level. The partial 16S rRNA gene sequence was deposited in GenBank (Accession MG517427.1). According to our laboratory team, this strain evidenced high ability to produce siderophores. The strain was grown in tubes containing 5 mL of Yeast Mannitol Broth (YMB) medium overnight at 26 °C in an orbital shaker (160 rpm) [11].

*Rhizobium* sp. strain E20-8 was previously isolated from the root nodules of *Pisum sativum* L. [14]. The 16S rRNA gene was amplified and the PCR products sequenced and used to identify the bacterium strain to genus level. The partial 16S rRNA gene sequence was deposited in GenBank (Accession:KY491644). *Rhizobium* sp. strain E20-8 was being capable of fixating N_2_ in symbiosis *P. sativum* L. [14] and producing indole-3-acetic acid [11]. The strain was grown in tubes containing 5 mL of Yeast Mannitol Broth (YMB) medium overnight at 26 °C in an orbital shaker (160 rpm) [15].

*Pseudomonas* sp. strain S22 was previously isolated from *Glycine max*. The 16S rRNA gene was amplified, the PCR products were sequenced and used to identify the bacterium strain to genus level, and they were deposited in GenBank (OM986010) *Pseudomonas* sp. strain S22 was used in field assays conducted by our laboratory and described as a plant growth promoter. The strain was grown in tubes containing 5ml of Tryptic Soy Broth (TSB) medium overnight at 26 °C in an orbital shaker (160 rpm) [16].

#### 2.2.2. Bacterium Tolerance to PE Microparticles

Bacteria were grown in 250 mL flasks with 10 mL of YMB medium supplemented or not with BIO (1% (*w*/*v*)) of different particle sizes (2.8 to 2 mm, 2 to 1 mm, 1 to 0.5 mm, 0.5 to 0.25 mm, and <0.25 mm) in a total of 6 conditions, including a control with no plastic included. Flasks were inoculated with 100 µL of a 10^8^ cells mL^−1^ culture grown as previously specified (Section 2.2.1). Optical density was measured at 600 nm, but due to the interference caused by the plastic material, the determination of protein content was used to determine bacterial growth. For that, the 10 mL of bacterial culture was centrifuged at 10,000 rpm for 10 min at 4 °C. The supernatant was discarded, and the pelleted cells were resuspended in 0.1 M phosphate buffer, pH 7.4, and sonicated using an ultrasonic processor (Vibra-Cell™ VCX 130, manufactured by Sonics & Materials, Danbury, CT, USA). Samples were sonicated in ice at 60% intensity for 30 s. Afterwards, sonication samples were centrifuged at 10,000 rpm for 10 min at 4 °C, and the supernatant was used to determine the protein content. For that, 50 µL of supernatant was transferred to a microplate well, and 250 µL of Biuret reagent was added [17]. After a 10 min incubation in the dark, the microplate was read at 540 nm in a Mobi™ Atomic Absorption Microplate Spectrophotometer (Gyeonggi, Republic of Korea). Results were expressed in mg/mL and used to estimate bacterial growth.

#### 2.2.3. Determination of BIO Microplastics Degradation Capacity

In order to ascertain the hypothetical ability of bacteria to degrade BIO microplastics, this compound was included in the growth medium as the sole source of carbon. For that analysis, 30 mL of minimal salt medium [18] were mixed with 0.1% (*w*/*v*) of <0.25 mm BIO microplastics. Tests were performed with *Bacillus* sp. strain J25, *E. ludwigii* sp. strain C11, and *Kosakonia* sp. strain O21 in medium with and without (controls) BIO microplastics. Each condition was replicated five-fold. Before inoculation, the medium containing BIO microplastics was sonicated in an ultrasonic bath (SONICA^®^ ultrasonic cleaner, SOLTEC Srl, Milan, Italy) to ensure the dispersion of BIO particles. Flasks were inoculated with 300 µL of 108-cell mL^−1^ fresh culture and grown during 30 days at 26 °C at 150 rpm. After the growth period, samples were centrifuged at 2000 rpm at room temperature for 5 min, allowing the deposition of bacterial cells but not the BIO microparticles. The pellet was resuspended in phosphate buffer (Section 2.2.2), and bacterial growth was estimated by the protein content, as described previously (Section 2.2.2). Results were expressed as mg protein/mL and growth compared between media with and without BIO microplastics.

### 2.3. In Planta Growth Assay with PE Microplastics

#### 2.3.1. Plant Species and Seed Preparation

Lettuce (*Lactuca sativa* L.) represents one of the most popular vegetables, with a global production of 27 million tonnes (2022 report, combined with chicory), with China alone producing 55% of the total produced worldwide (FAO 2022). Since lettuce is a cool-season vegetable crop with an optimal air temperature of 23 °C and an optimal root temperature of 19 °C [19], production systems and agronomic practices have a major impact on the yield and quality characteristics of lettuce [20], with mulching being frequently used because it promotes early production and higher yields [21].

*L. sativa* seeds (Hilde III variety, Alípio e Irmão, Porto, Portugal) were disinfected in 70% ethanol for 1 min and then transferred to a 25% bleach solution for 12 min under agitation. Then, the seeds were transferred to a distilled water solution for 3 min, this last stage being repeated 4 times. The disinfected seeds were then placed in petri dishes with Murashige and Skoog medium [22] at half strength. After germination, seedlings were ready to be transplanted to soil.

#### 2.3.2. Soil Preparation

Artificial OECD was used (OECD 208). According to a previous study, the proportions of microplastics found in a field after 5 years of continuous mulching were 33.49% of microparticles <1 mm, 32.49% between 1 mm and 3mm, and 34.02% >3 mm [23]. Thus, in this study, a mixture of equal proportions of particles <1 mm, 1 mm to 2.8 mm, and >2.8 mm was used. Soil and microplastics were combined to generate conditions with different BIO concentrations (0.2%, 0.5%, 1%, and 5% *w*/*w*), and a control without BIO microplastics was also included. The soil–BIO microplastics mixtures were autoclaved separately.

#### 2.3.3. Plant Growth Assay

Containers (150 mL) were filled with 100 g of soil (no BIO microplastics) or a plastic/soil combination. In each container, an *L. sativa* seedling (Section 2.3.1) was planted. A total of 5 replicates × 5 conditions (4 BIO concentrations + no BIO microplastics) × (1 bacterial strain + no inoculation)) were performed, totalling 50 containers. Inoculated controls received 1 mL of *Kosakonia* sp. strain O21 culture (108 cells mL^−1^). Non-inoculated conditions received 1 mL growth medium. After inoculation, the containers were watered with distilled water every day in order to maintain 60% water holding capacity (WHC) until the experiment was completed. Plants were cultivated in greenhouse conditions for 14 days under artificial light (1450 μmol/m^2^/s) and a photoperiod of 12 h and 26 ± 1 °C and 18 ± 1 °C during light and dark periods, respectively.

At the end of the growth period, plants were collected. The roots were rinsed in tap water and then in deionized water to remove substrate particles. The roots were separated from shoots. The fresh weights were determined. Samples for photosynthetic pigments were used immediately, while samples for other biochemical parameters were lyophilized for 72 h, weighted to obtain the dry weight, and immediately frozen (−20 °C) until needed. Dry weight was expressed in mg plant^−1^.

#### 2.3.4. Photosynthetic Pigments

Fresh shoot samples were homogenized with a pestle and mortar in 80% cold acetone (1:2 *w*/*v*) and rested for 45 min in the dark. Extracts were centrifuged at 4000× *g* for 5 min, and pigment content was measured using the method published by Wellburn and Lichtenthaler [24]. Wellburn and Lichtenthaler’s (1984) formulae (Chlorophyll a (mg/g) = 12.7(A663) − 2.69(A645); Chlorophyll b (mg/g) = 22.9(A645) − 4.68(A663); Carotenoid (mg/g) = A480 + 0.114(A663) − 0.638(A645)) were used to determine chlorophylls a and b, as well as carotenoids, based on absorbance measurements at 663 nm, 645 nm, and 480 nm. The results were represented as micrograms per gram of dry weight (μg/g DW) [16].

#### 2.3.5. Biochemical Parameters

##### Extraction

To frozen lyophilized roots and shoots, sodium phosphate buffer (50 mM sodium dihydrogen phosphate monohydrate, 50 mM disodium hydrogen phosphate dihydrate, 1 mM ethylenediaminetetraacetic acid disodium salt dihydrate (EDTA), 1% (*v*/*v*) Triton X-100, 1% (*v*/*v*) polyvinylpyrrolidone (PVP), 1 mM dithiothreitol (DTT), pH 7.0) was added (1:2 *w*/*v*), and then samples were lysed using an ultrasound probe (Vibra Cell Ultrasonic Processor, Sonics, Newtown, CT, USA) for 60 s, utilizing a continuous cycle with 50% amplitude. The samples were centrifuged at 10,000× *g* for 10 min. The supernatant was collected and stored at −20 °C for superoxide dismutase (SOD), catalase (CAT), and glutathione S-Transferase (GST) activity and for protein, protein carbonylation (PC), and lipid peroxidation (LPO) content. For electron transport system (ETS) activity, the process was similar, but samples were centrifuged at 3000× *g* for 3 min and determination was performed immediately after centrifugation [25].

##### Electron Transport System

ETS activity was measured using the King and Packard [26] method, with modifications by Owens and King (1975). To 37.5 μL of supernatant, 107 μL of BSS buffer (0.13 M Tris-HCl, 0.3% (*v*/*v*) Triton X-100, pH 8.5), 35.7 μL of NAD(P)H (1.7 mM NADH and 250 μM NADPH), and 71.4 μL of 8 mM p-IodoNitroTetrazolium (INT) were added. The absorbance was measured at 490 nm for 10 min at 25 s intervals. The amount of formazan produced was determined using its molar extinction coefficient (15,900 M^−1^ cm^−1^) and presented in micromolar formazan per minute per gram of dry weight (µmol/min/g DW) [16].

##### Protein Content

The protein content was determined using the method published by Robinson and Hogden [17]. To 50 μL of supernatant, 250 μL of biuret reaction solution was added. Samples were incubated in the dark at room temperature for 10 min. The absorbance was measured at 540 nm using bovine serum albumin (BSA) as a reference (5–40 mg/mL). The results were represented as milligrams of protein per gram of dry weight (mg prot/g DW) [16].

##### Protein Carbonylation

To determine protein carbonylation, the method described by Mesquita et al. [27] was employed. In a 96-well plate, 125 µL of supernatant were mixed with 120 µL of 10 mM 2,4-dinitrophenylhydrazine (DNPH) and incubated for 10 min at room temperature. Next, 60 µL of 6 M sodium hydroxide (NaOH) were added and incubated for another 10 min at room temperature. Absorbance was measured at 450 nm with 10 s of agitation before measurement. The molar extinction coefficient of protein carbonyl-DNPH hydrazine (ε = 22,308 M^−1^ cm^−1^) was used to calculate the protein carbonylation and expressed in nanomoles of carbonyl groups per gram of dry weight (nmol/g DW) [25].

##### Superoxide Dismutase

The activity of SOD was measured by converting nitroblue tetrazolium (NBT) to NBT diformazan using the Beauchamp and Fridovich method [28]. To 25 μL of supernatant, 25 μL of 51.6 mU/mL xanthine oxidase and 250 μL of reaction buffer (50 mM Tris-HCl (pH 8.0), 0.1 mM Diethylenetriaminepentaacetic acid (DTPA), 0.1 mM Hypoxanthine) containing 68.4 µM NBT were added and incubated for 20 min at room temperature with rotation. The absorbance was measured at 640 nm. One unit of enzymatic activity (U) represents a 50% reduction of NBT. The results were expressed as U per gram of dry weight (U/g DW) [16].

##### Catalase (CAT)

The protocol of Johansson and Borg [29] was used to assess catalase activity. In a 96-well plate, 25 µL of supernatant was mixed with 125 µL of 35.28 mM reaction buffer (potassium phosphate 50 mM, pH 7.0, EDTA 1 mM, Triton X-100 1%), 37.5 µL methanol, and 25 µL hydrogen peroxide (H_2_O_2_) and incubated for 20 min at room temperature in an orbital shaker (150 rpm). After adding 37.5 µL of 10 M potassium hydroxide (KOH) and 37.5 µL of 34.2 mM Purpald, a second period of 10 min incubation under the same conditions was performed. Finally, 12.5 µL of 65.2 mM potassium periodate was added and incubated for 10 more min at the same conditions. The absorbance was measured at 540 nm, using formaldehyde as a reference (2.5–20 µg/mL). The results are presented as milliunits of enzymatic activity (mU) per gram of fresh weight (mU/g FW) [25].

##### Soluble Carbohydrates

The soluble carbohydrate content was determined using the method reported by Dubois et al. [30], with some modifications. To a 15 µL sample, 900 µL of 98% sulfuric acid and 150 µL of 5% phenol were added. The mixture was then incubated for one hour at room temperature. The samples were centrifuged at 10,000× *g* for 5 min, the supernatant was collected, and absorbance was measured at 492 nm. Glucose was used as reference (1–10 mg/mL). The results were presented as milligrams of glucose per gram of dry weight (mg/g DW) [16].

##### Lipid Peroxidation

Lipid peroxidation was determined using the method of Buege and Aust [31] for estimating cell membrane damage based on lipid peroxidation levels. In a 2 mL microtube, the pellet samples were combined with 200 µL of 0.5% thiobarbituric acid (TBA) and 150 µL of 20% trichloroacetic acid (TCA) and incubated at 96 °C for 25 min. The reaction was stopped by placing the microtubes in ice. The cooled liquid was pipetted into a 96-well plate, and its absorbance was read at 532 nm. Lipid peroxidation was calculated using the malondialdehyde (MDA) extinction coefficient (ε = 1.56 × 105 M^−1^ cm^−1^) and expressed in nanomoles MDA equivalents per gram of dry weight (nmol MDAeq/g DW) [11].

##### Glutathione S-Transferase (GST)

Glutathione S-transferase activity was determined using the approach given by Habig [32]. In a 96-well plate, 100 µL of supernatant were mixed with 200 µL of reaction solution (potassium phosphate buffer 0.1 M, pH 6.5, 10 mM reduced glutathione (GSH), and 60 mM 1-chloro-2,4-dinitrobenzene (CDNB)). Absorbance was measured at 340 nm for 5 min at 15-s intervals, with agitation lasting 6 s before reading. The results were presented as milliunits of enzymatic activity (mU) per gram of dry weight (mU/g DW) [25].

##### Statistical Analysis

Hypothesis testing was done on every parameter evaluated. GraphPad Prism 8.0.2 for Windows (GraphPad Software, Boston, MA, USA) was used to do a two-way analysis of variance (ANOVA) and then a Tukey’s test. Asterisks were used to indicate differences between inoculated and non-inoculated plants (Sidak’s test) at each concentration of BIO microplastics (0%, 0.2%, 0.5%, 1%, and 5%), and different letters were used to indicate differences among BIO concentrations (lowercase for non-inoculated and uppercase for inoculated plants). Significant differences were only considered when the *p*-value was less than 0.05. A Euclidean distance similarity matrix for roots and shoots was computed using biochemical parameter data. After these similarity matrices were made, principal coordinates (PCOs) were used for ordination analysis. The PCO graph included biochemical parameter Pearson correlation vectors (correlation ≥ 0.5) as supplementary variables, which made it possible to determine which descriptors were primarily responsible for the variations among the conditions that were evaluated.

## 3. Results

### 3.1. Characterization of BIO Microparticles

#### 3.1.1. Distribution of Microparticles by Size

**Figure 1:**
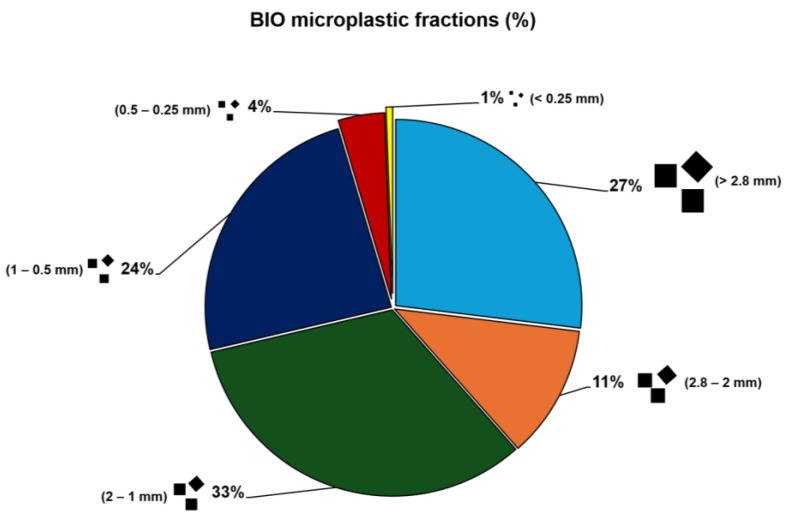
Proportion of biodegradable (BIO) microparticles with different sizes after production. Microparticles were separated by size in 6 fractions: >2.8 mm; 2.8–2 mm; 2–1 mm; 1–0.5 mm; 0.5–0.25 mm; and <0.25 mm.

In the accounted biodegradable plastic fractions (Figure 1), the highest contributors were size categories of 2–1 mm (33%), >2.8 mm (27%), 1–0.5 mm (24%), and 2.8–2 mm (11%). The lowest contributors were the size categories of 0.5–0.25 mm (4%) and <0.25 mm (1%).

#### 3.1.2. SEM-EDS Analysis of Microparticles

**Figure 2:**
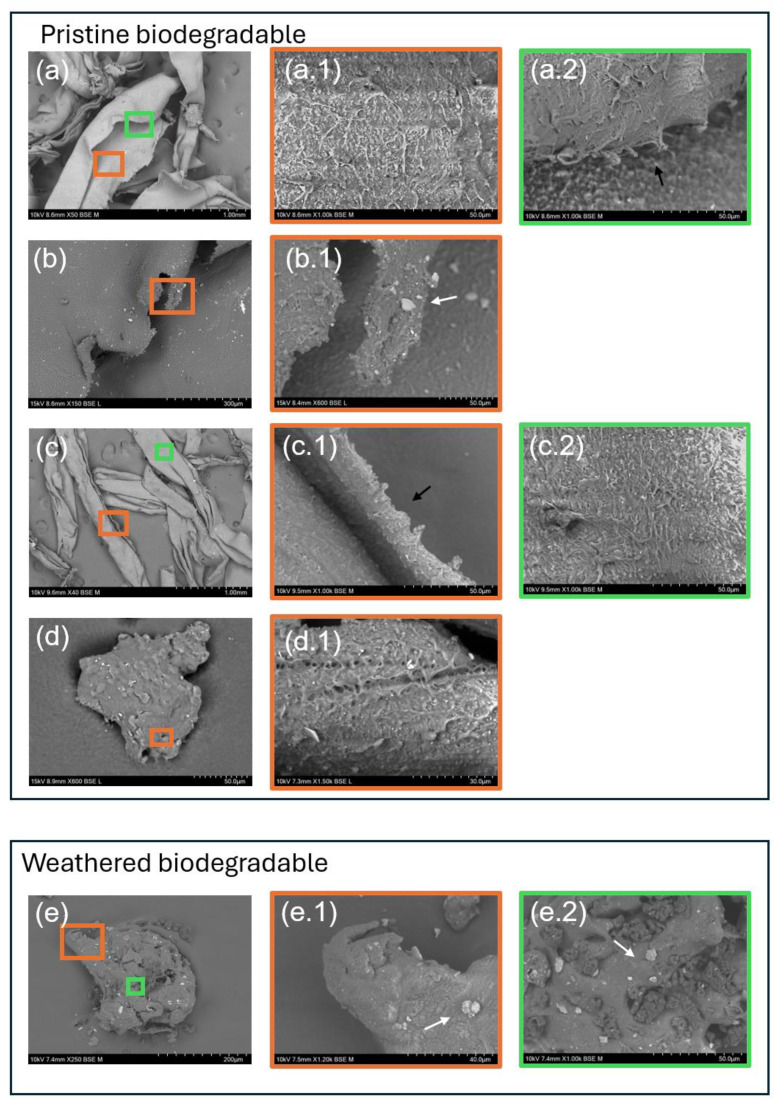
SEM images of biodegradable (BIO) microplastics; (**a**) BIO 2–1 mm (50×); (**a.1**) BIO 2–1 mm (1000×); (**a.2**) BIO 2–1 mm (1000×)—View with protuberances; (**b**) BIO 1–0.5mm (150×); (**b.1**) BIO 1–0.5 mm (600×)—Detail of a protuberance; (**c**) BIO 0.5–0.25 mm (40×); (**c.1**) BIO 0.5–0.25 mm (1000×)—Detail of the edge; (**c.2**) BIO 0.5–0.25mm (1000×)—detail of the surface; (**d**) BIO < 0.25 mm (600×); (**d.1**) BIO < 0.25 mm (1500×); (**e**) BIO UV (250×); (**e.1**) BIO UV (1200×)—Edge of the particle; (**e.2**) BIO UV (1000×)—Surface of the particle. White arrows indicate silicate particles (confirmed by EDS); black arrows indicate plastic protuberances.

A scanning electron microscope (SEM) was used to examine the surface morphology of microplastics (Figure 2). The size intervals in which they were grouped displayed varying textures on the surface of the microplastics from mulching films. These textures ranged from smooth to rough, with pits, flakes, grooves, and attached particles. The BIO microplastics presented fibre-like forms at larger size intervals as well, but they have a more rugged texture made up of many sharp protrusions. This feature is present in all the BIO microplastics that are not weathered. When BIO plastics were subjected to UV weathering, they appeared to have a smoother texture than those not weathered.

#### 3.1.3. ATR-FTIR Analysis of Microparticles

**Figure 3:**
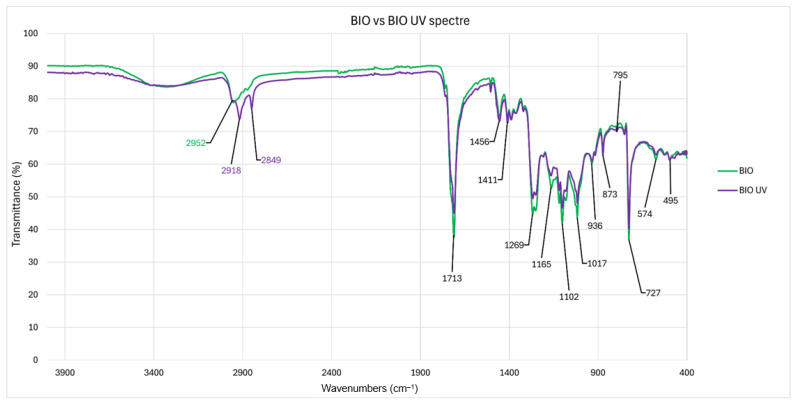
ATR-FTIR spectra of BIO microplastics and weathered BIO microplastics.

The biodegradable plastic film’s ATR-FTIR spectra (Figure 3) show the presence of a large peak between 3000 and 3600 cm^−1^, which may be connected to the stretching vibrations of hydroxyl groups that are frequently found in starch structures [33]. Bands of symmetric and asymmetric stretching C–H vibrations were seen at 2847 and 2917 cm^−1^ [34]. Strong vibrations of C=O bonds stretching in the ester groups of PBAT (polybutylene adipate terephthalate) were found at 1712 cm^−1^ [35]. The spectra exhibit several smaller bands consistent with the vibrations of C–C, C–O, C–H, C–N, and N–H bonds in the 1000–1500 cm^−1^ range [36]. Among these bands is a typical 1270 cm^−1^ for PBAT and 1020 cm^−1^ for cellulose [37,38]. A signal was detected at 726 cm^−1^, which was associated with the existence of a hydrocarbon chain consisting of four or more successive methylene groups [39]. Weathering, or peaks at 2918 cm^−1^ and 2850 cm^−1^, related to C–H stretch, which is known to be formed after blending of PBAT with starch or cellulose, became visible, pointing to the potential occurrence of crosslinking [40,41].

#### 3.1.4. Thermogravimetric Analysis

**Figure 4:**
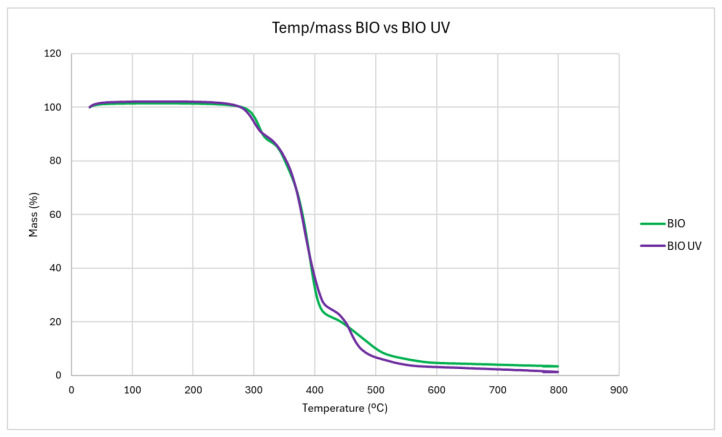
Thermogravimetric analysis of BIO microplastics and weathered BIO microplastics.

The BIO thermograms are represented either without or with weathering, as can be seen in Figure 4. Only one degradation step is represented for each material, as is characteristic of nearly all polymers.

In both weathered and untreated BIO microplastics, degradation starts at about 280 °C and is complete at 518 °C, showing the most important weight loss between 285 °C and 400 °C, followed by a second weight loss from 440 °C to 500 °C. Yet, in BIO microplastics exposed to weathering, the TG data present evidence that on the temperature frame from 440 °C to 500 °C, the degradation seems to be more acute than in the non-exposed counterpart. Stablishing 95% of the mass lost on thermal degradation as a reference, with PE-UV, this threshold is achieved at 480 °C, while for PE-UV, it is registered as happening at 500 °C.

#### 3.1.5. Zeta Potential

The surface charge for BIO microplastics was found to be −23.8 (mV) based on the zeta values. The surface charge of weathered BIO microplastics was not obtained since insufficient weathered material was available for sieving in order to obtain particles suitable for zeta potential measurements.

### 3.2. Bacterium Tolerance to BIO

**Figure 5:**
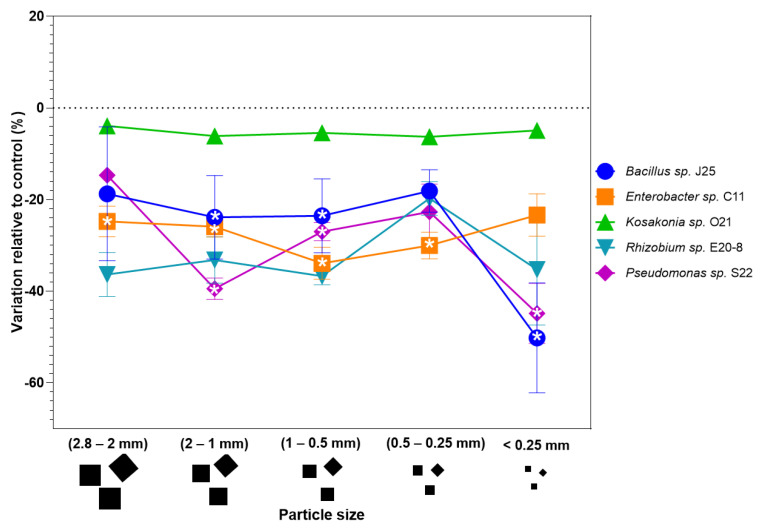
Bacterial growth. Effect of BIO microplastics exposure on protein content of distinct bacterial strains. *Bacillus* sp. J25 (blue circles), *Enterobacter ludwigii* sp. C11 (orange squares), *Kosakonia* sp. O21 (green triangles), *Rhizobium* E-20-8 (teal inverted triangles), and *Pseudomonas* sp. S22 (purple diamonds). BIO concentration of 1% (*w*/*v*). Particle sizes are grouped by the intervals of 2.8–2 mm; 2–1 mm; 1–0.5 mm; 0.5–0.25 mm; and <0.25 mm. Values are means of five replicates + standard error. Growths significantly different (*p* < 0.05) from the respective control (same bacterial strain not exposed to BIO microplastics) are marked with an asterisk.

Research on the effect of BIO particle size on protein content (as a measure of bacterial growth) was carried out across the strains *Bacillus* sp. J25, *Enterobacter* sp. C11, *Kosakonia* sp. O21, *Rhizobium* sp. E-20-8, and *Pseudomonas* sp. S22. Figure 5 depicts the results as a percentage difference in protein content compared to a control group (bacteria non-exposed to BIO) for various particle sizes.

*Bacillus* sp. J25 demonstrated a consistent reduction of about −20% in protein content regardless of particle size, with the exception of the smaller particle size (<0.25 mm), which had a more pronounced reduction of about −50% compared to control. In *Enterobacter ludwigii* sp. C11, protein variation remained between −20% and −40% across all particle sizes, with an increasingly smaller negative variation compared to control, as the particle size gets smaller, and at BIO < 0.25 mm the difference is not statistically significant from the control.

*Kosakonia* sp. O21 had the most constant protein content, with growth reductions being smaller than 5% and non-significant compared to the control. This bacterial strain was the least impacted by particle size or microplastics exposure.

*Rhizobium* sp. E-20-8 was the most affected strain by larger particle sizes (decreases around 35%), but tolerance to smaller particles was higher, especially for 0.5–0.25 mm particles (decreases around 20%). Nevertheless, differences were not statistically significant.

*Pseudomonas* sp. S22 had the most variable response to particle size, with variation in protein content relative to control being higher at 2–1 mm and <0.25 nm particle sizes (between −40% to −50%) and being lower at 0.5–0.25 mm (around −20%).

### 3.3. Bacterial Ability to Degrade BIO Microparticles

The ability to use BIO microplastics as a carbon source was evaluated in the three strains that were assessed as being less affected by BIO microparticles (*Enterobacter* sp. strain C11, *Kosakonia* sp. strain O21, and *Bacillus* sp. strain J25), with protein content determination being the key metric for growth analysis.

**Figure 6:**
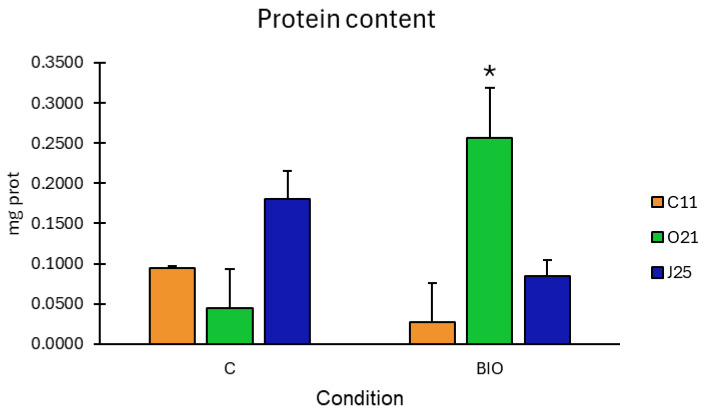
Bacterial growth with BIO microplastics as sole source of carbon. Growth estimated by protein content of *Bacillus* sp. J25 (blue), *Enterobacter ludwigii* sp. C11 (orange), and *Kosakonia* sp. O21 (Green) exposed to minimal medium (C) or supplemented with 0.1% BIO particles with size <0.25 mm (BIO). Values are means of five replicates + standard error. Growths significantly different (*p* < 0.05) from the respective control (same bacterial strain not exposed to BIO) are marked with an asterisk.

*Kosakonia* sp. O21 exhibited a substantial and significant increase in protein content, with a 5-fold increase compared to the control (Figure 6). Both *Enterobacter* sp. C11 and *Bacillus* sp. J25 did not show significant growth between the control and the test group with the BIO microplastics.

### 3.4. Influence of Kosakonia sp. O21 on Growth and Biochemistry of Plants Exposed to BIO Microplastics

#### 3.4.1. Plant Growth

**Figure 7:**
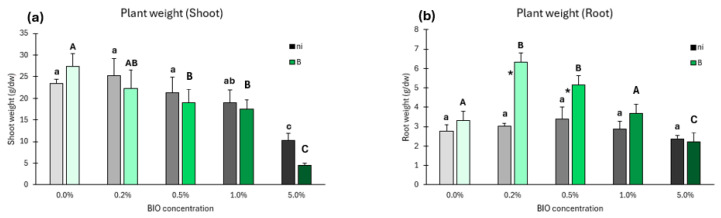
Plant growth. Shoot (**a**) and root (**b**) growth of lettuce plants not inoculated or inoculated with *Kosakonia* sp. O21 grown at different concentrations of biodegradable (BIO) microplastics. Ni—no bacterial inoculation; B—inoculation with bacteria. Values are means of at least five replicates ± standard error. Different uppercase letters indicate significant differences (*p* < 0.05) among conditions in plants inoculated with bacteria, different lowercase letters indicate significant differences among conditions in plants not inoculated, and asterisks indicate significant differences between inoculated and not inoculated plants for the same condition.

Evident differences across concentrations of BIO microplastics were present for both shoot and root growth in non-inoculated plants (ni). The shoot weight of non-inoculated plants (Figure 7a) was higher at BIO 0.2%, and an increase in the concentration of BIO resulted in the decrease in shoot weight, only significant at BIO 5%. In inoculated plants (B), the shoot weight also demonstrated a general trend of reduced shoot weight with the increase in BIO concentration, significant at 0.5%, 1%, and 5%. At each BIO concentration, no significant differences between non-inoculated and inoculated plants were observed.

Roots of non-inoculant plants followed a different trend of shoots (Figure 7b), with root weight not being significantly affected by BIO concentrations. The roots of inoculated plants were affected by BIO microplastics, with significant increases at BIO 0.2% and 0.5% and a significant decrease at 5% relative to control. At each BIO concentration, significant differences between inoculated and non-inoculated plants were observed at BIO 0.2% and 0.5%, with an increased root weight of the inoculated plants in relation to the non-inoculated ones.

#### 3.4.2. Root Biochemistry

##### Protein Content

**Figure 8:**
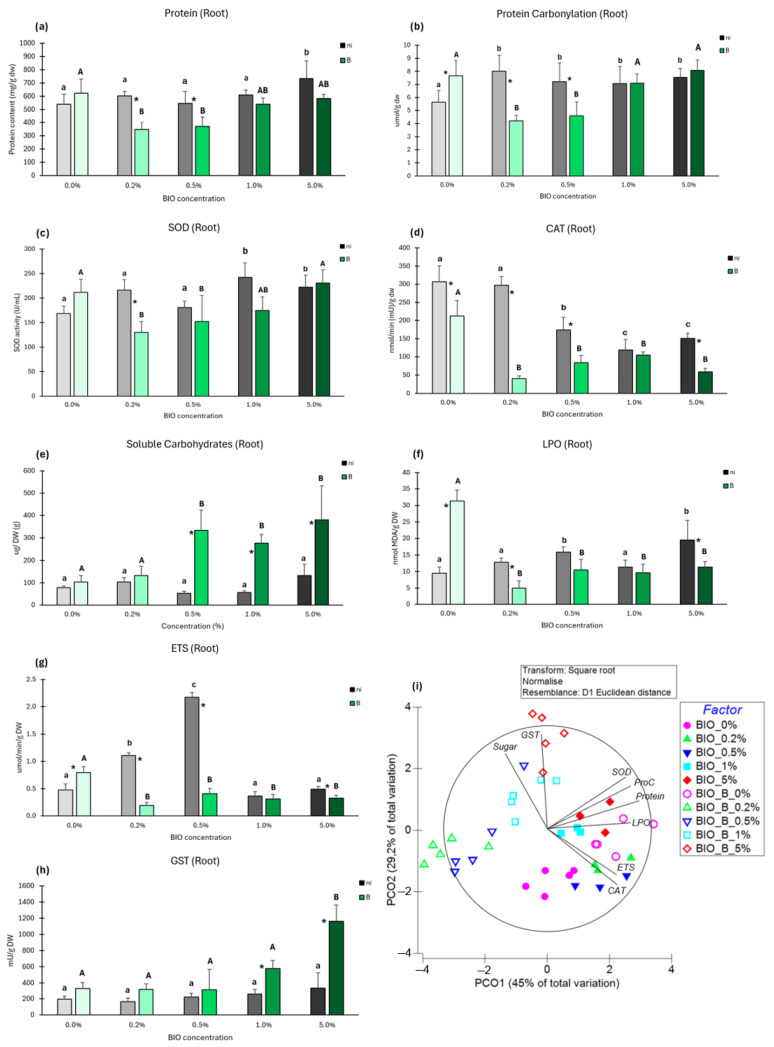
Biochemical parameters of the roots of not inoculated (dark bars) or inoculated (green bars) lettuce plants grown with biodegradable (BIO) microplastics at different concentrations (0% to 5%) in different inoculation conditions (Ni—no bacterial inoculation; B—bacterial inoculation with *Kosakonia* sp. O21); (**a**) Protein content; (**b**) Protein Carbonylation; (**c**) Superoxide dismutase activity (SOD); (**d**) Catalase activity (CAT); (**e**) Soluble carbohydrates; (**f**) Lipid peroxidation (LPO); (**g**) electron transport system activity (ETS); (**h**) Glutathione S-transferase (GST); (**i**) Principal coordinates ordination of biochemical parameters in the roots of inoculated and non-inoculated plants. Values are means of five replicates + standard error. Different lowercase letters indicate significant differences (*p* < 0.05) among conditions in non-inoculated plants, different uppercase letters indicate significant differences among conditions in inoculated plants, and asterisks indicate significant differences between inoculated and non-inoculated plants for the same BIO concentration.

Only at 5% BIO was the protein content of roots from non-inoculated plants significantly changed (Figure 8a). In inoculated plants, proteins were significantly reduced at BIO 0.2% and 0.5% relative to the control. For the same BIO concentration, the protein content was significantly lower in inoculated plants at BIO 0.2% and 0.5%.

##### Protein Carbonylation

The exposure to BIO microparticles significantly increased protein carbonylation in the roots of non-inoculated plants (Figure 8b) compared to the control. In inoculated plants, a significant reduction was observed at BIO 0.2% and 0.5%. For the same BIO concentration, significant differences in protein carbonylation were observed between inoculated and non-inoculated plants at BIO 0%, 0.2%, and 0.5%.

##### Superoxide Dismutase (SOD)

The presence of BIO microparticles significantly increased SOD activity in the roots of non-inoculated plants at higher concentrations (Figure 8c) compared to the control. In inoculated plants, SOD activity significantly decreased at BIO 0.2% and 0.5%. For the same BIO concentration, significant differences in SOD activity were observed between inoculated and non-inoculated plants at BIO 0.2%.

##### Catalase (CAT)

Activity was significantly lower in the roots of non-inoculated plants exposed to BIO microparticles in the 0.5–5% range (Figure 8d) compared to the control. In inoculated plants, significant changes in CAT activity were observed compared to the control in all BIO concentrations, with 2.1- to 4.2-fold reductions. For the same BIO concentration, significant differences in CAT activity were observed between inoculated and non-inoculated plants in the presence of BIO 0%, 0.2%, 0.5%, and 5%.

##### Soluble Carbohydrates

Exposure to BIO microparticles did not influence significantly soluble carbohydrate content relative to control (Figure 8e). In inoculated plants, no significant changes were observed between BIO 0% and 0.2%, but increases between 3.2- and 3.5-fold were observed in higher concentrations when compared to the control. When comparing inoculated and non-inoculated plants for the same BIO concentration, significant differences in soluble carbohydrates were only found at BIO 0.5%, 1%, and 5%.

##### Lipid Peroxidation

Significant increases in LPO content were observed in the roots of non-inoculated plants exposed to BIO 0.5% and 5% compared to the control (Figure 8f). In inoculated plants, significant decreases (3.2- to 6.4-fold) in LPO were observed across all BIO concentrations when compared to control. Comparing inoculated and non-inoculated plants at the same BIO concentration, at BIO 0% inoculated plants presented significantly higher LPO levels, but at BIO 0.2% and 5% LPO was lower in inoculated plants.

##### Electron Transport System (ETS)

BIO microparticles increased ETS activity in the roots of non-inoculated plants at BIO 0.2% and 0.5% (Figure 8g). In inoculated plants, all BIO concentrations significantly reduced ETS activity compared to the control. Comparing inoculated and non-inoculated plants for the same BIO concentration, significant differences in ETS activity were found at BIO 0% (increase) and at 0.2%, 0.5%, and 5% (decreases).

##### Glutathione S-Transferase

All concentrations of BIO microparticles did not cause any significant changes in GSTs activity in the roots of non-inoculated plants (Figure 8h). In inoculated plants, an increasing trend in GST activity with BIO concentration was observed that was only significant at BIO 0.5%. Comparing inoculated and non-inoculated plants at the same BIO concentration, significant differences in GSTs activity were found at the higher BIO concentrations (1% and 5%).

##### Multivariate Analysis

From the multivariate analysis of root biochemical changes induced by the factors studied (BIO exposure and inoculation with *Kosakonia* sp. O21), it is possible to observe that most biochemical markers were strongly correlated with PCO1 and therefore more related to the exposure to BIO microplastics and their concentrations, confirming that the presence of BIO microplastics is the factor inducing most of the biochemical changes. However, inoculation with *Kosakonia* sp. O21 also contributed to biochemical changes in roots. Bacterial inoculation under BIO microplastics exposure strongly correlated with the negative side of the PCO1 axis and both the positive (BIO 1% and 5%) and negative (BIO 0.2% and 0.5%) sides of the PCO2 axis, evidencing that under BIO microplastics exposure, the non-inoculated plants triggered mechanisms (SOD and CAT), increased the metabolic activity (ETS) and the protein content, and, at the higher BIO concentrations (1% and 5%), correlation with cell damage (LPO and PC) was also strong. Root cells of plants exposed to lower concentrations of BIO microplastics (0.2%, 0.5%) were not correlated with any biochemical parameter, and those exposed to higher BIO concentrations (1% and especially 5%) strongly correlated with GSTs activity and sugar content.

#### 3.4.3. Shoot Biochemistry

##### Photosynthetic Pigments

**Figure 9:**
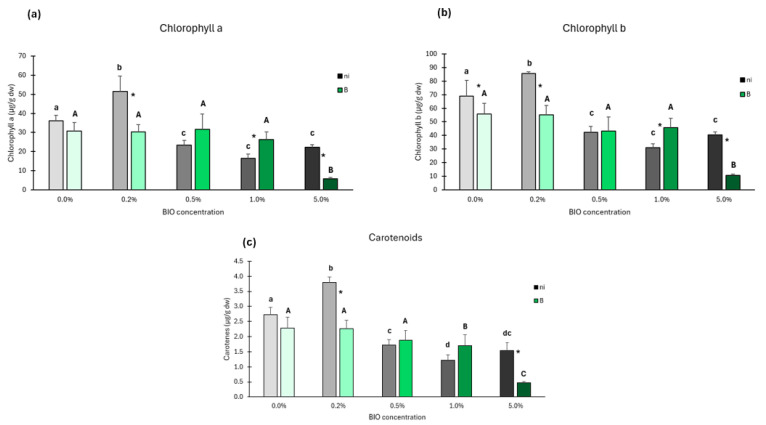
Photosynthetic pigments of the shoots of not inoculated (dark bars) or inoculated (green bars) lettuce plants grown with biodegradable microplastics (BIO) at different concentrations (0% to 5%) in different inoculation conditions (Ni—no bacterial inoculation; B—bacterial inoculation with *Kosakonia* sp. O21); (**a**) Chlorophyll a; (**b**) Chlorophyll b; (**c**) Carotenoids. Values are means of five replicates + standard error. Different lowercase letters indicate significant differences (*p* < 0.05) among conditions in non-inoculated plants, different uppercase letters indicate significant differences among conditions in inoculated plants, and asterisks indicate significant differences between inoculated and non-inoculated plants for the same BIO concentration.

The presence of BIO microparticles changed chlorophyll a content. At BIO 0.2%, a significant increase is observed; for higher BIO concentrations, significant decreases were noticed compared to control (Figure 9a). In inoculated plants, the chlorophyll a content only changed significantly at BIO 5%, a 3-fold decrease being observed compared to control. For the same BIO concentration, chlorophyll a was significantly higher in non-inoculated plants at 0.2% and 5%, and lower at 1%.

Chlorophyll b followed the same trend of chlorophyll a, significantly increasing at BIO 0.2% and decreasing at higher BIO concentrations relative to BIO 0% (Figure 9b). At inoculated conditions, changes were also similar to chlorophyll a, with only 5% BIO inducing a significant decrease in chlorophyll b compared to the inoculated control. For the same BIO concentration, chlorophyll b was significantly higher in non-inoculated plants at BIO 0%, 0.2%, and 5%, and lower at BIO 1%.

Exposure to BIO microparticles significantly increased carotenoids in non-inoculated plants at BIO 0.2% (Figure 9c), but significant decreases were observed for higher BIO concentrations (0.5%, 1%, and 5%). At inoculated conditions, lower BIO concentrations (0.2% and 0.5%) did not significantly influence carotenoids content, but at BIO 1% and 5%, significant decreases were observed, especially at 5%. At the same BIO concentration, carotenoids were higher in non-inoculated compared to inoculated plants for BIO 0.2% and 5%.

##### Protein Content

**Figure 10:**
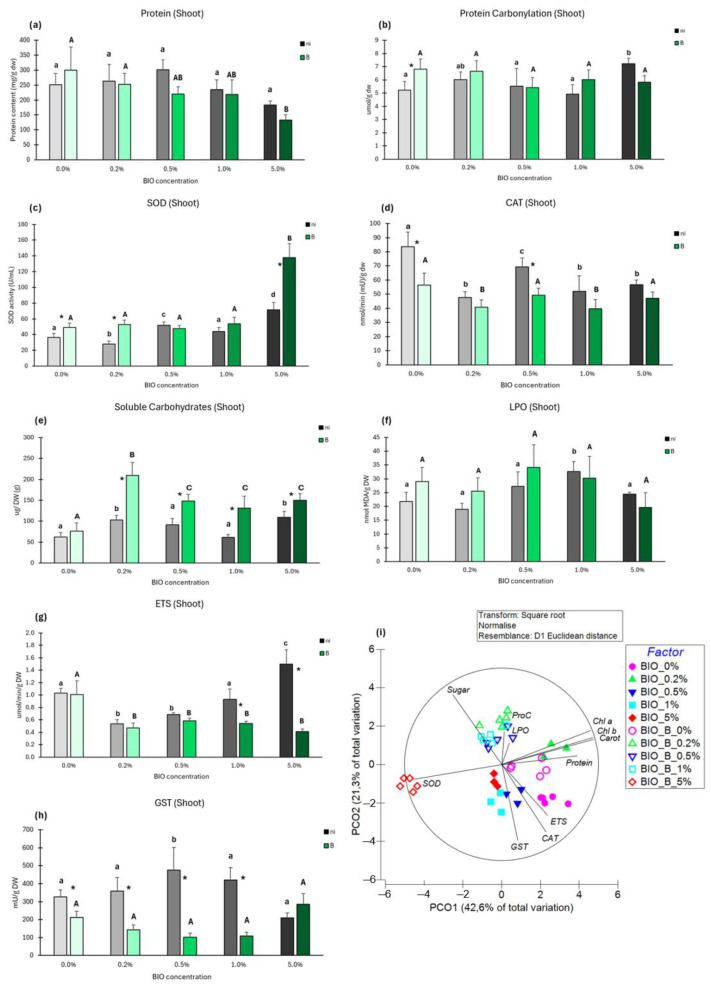
Biochemical parameters of the shoots of not inoculated (dark bars) or inoculated (green bars) lettuce plants grown with biodegradable microplastics (BIO) at different concentrations (0% to 5%) in different inoculation conditions (Ni—no bacterial inoculation; B—bacterial inoculation with *Kosakonia* sp. O21); (**a**) Protein content; (**b**) Protein Carbonylation; (**c**) Superoxide dismutase activity (SOD); (**d**) Catalase activity (CAT); (**e**) Soluble carbohydrates; (**f**) Lipid peroxidation (LPO); (**g**) electron transport system activity (ETS); (**h**) Glutathione S-transferase (GST); (**i**) Principal coordinates ordination of biochemical parameters in the roots of inoculated and non-inoculated plants. Values are means of five replicates + standard error. Different lowercase letters indicate significant differences (*p* < 0.05) among conditions in non-inoculated plants, different uppercase letters indicate significant differences among conditions in inoculated plants, and asterisks indicate significant differences between inoculated and non-inoculated plants for the same BIO concentration.

BIO microparticles did not significantly change the protein content of shoots from non-inoculated plants (Figure 10a). In inoculated plants, a declining trend in protein content is observed, but significant variation relative to control was only observed at BIO 5%. Comparing the same BIO concentration, no significant variation was found between inoculated and non-inoculated plants.

##### Protein Carbonylation

Only 5% BIO significantly affected the PC level in the shoots of non-inoculated plants (Figure 10b). No significant changes in PC levels were observed among inoculated plants. For the same BIO concentration, significant differences in PC content were only observed between inoculated and non-inoculated plants at BIO 0%.

##### Superoxide Dismutase (SOD)

BIO microparticles both reduced (0.2%) and increased (0.5% and 5%) SOD activity significantly compared to the non-inoculated control (Figure 10c). In inoculated plants, only exposure to BIO 5% significantly changed SOD activity (2.8-fold increase) relative to the control. For the same BIO concentration, significantly higher SOD activity was observed in inoculated compared to non-inoculated plants at BIO 0%, 0.2%, and 5%.

##### Catalase (CAT)

Exposure to all BIO concentrations significantly decreased CAT activity in the shoots of non-inoculated plants (Figure 10d). In inoculated plants, significant decreases in CAT activity were observed at BIO 0.2% and 1% compared to control. For the same BIO concentration, non-inoculated plants evidenced higher CAT activity in shoots than inoculated ones at BIO 0% and 0.5%.

##### Soluble Carbohydrates

BIO microparticles significantly increased the soluble carbohydrate content in the shoots of non-inoculated plants at BIO 0.2% and 5% (Figure 10e). In inoculated plants, significant increases were observed at all BIO concentrations, the highest content being recorded at BIO 0.2% (2.8-fold). For the same BIO concentration, inoculated plants presented significantly higher content in soluble carbohydrates than their non-inoculated counterparts from BIO 0.2% to 5%.

##### Lipid Peroxidation

BIO microparticles had little effect on the LPO content of shoots both from inoculated and non-inoculated plants (Figure 10f). The only significant alteration was the higher LPO level in non-inoculated plants exposed to 1% BIO. For the same BIO concentration, no significant differences were observed between inoculated and non-inoculated plants.

##### Electron Transport System (ETS)

Only BIO 1% did not significantly affect the ETS activity in the shoots of non-inoculated plants (Figure 10g). At BIO 0.2% and 0.5%, significant decreases relative to the control were observed, and at BIO 5%, there was an increase of 1.6-fold. In inoculated plants, significant changes in ETS activity were observed at all BIO concentrations (0.2% to 5%), as significant decreases were noticed compared to the control. Significant differences for the same BIO concentration in ETS activity were found at BIO 1% and 5%.

##### Glutathione S-Transferase (GST)

BIO microparticles significantly increased GSTs activity in the shoots of non-inoculated plants at 0.5% compared to the control (Figure 10h). In inoculated plants, no significant changes in GSTs activity were observed at any BIO condition (0% to 5%). For the same BIO concentration, no differences were observed at BIO 5% between inoculated and non-inoculated plants, but in all other concentrations (0% to 1%), non-inoculated plants presented higher GSTs activity than inoculated ones.

##### Multivariate Analysis

From the PCO diagram of the biochemical changes induced by the factors studied (BIO exposure and inoculation with *Kosakonia* sp. O21) on lettuce shoots, PCO1 represents 42.6% of total variation and separates controls (both inoculated and non-inoculated) and BIO 0.2% non-inoculated on the more positive side of axis 1 from the BIO 5% inoculated on the far negative side of axis 1 and from the rest of the conditions near the origin of axis 1. PCO2 represents 21.7% of total variation and separates some inoculated conditions (BIO 0.2%, 0.5%, and 1%) from the other conditions near the origin (BIO 0.2% non-inoculated and BIO 0% inoculated) or on the negative side (BIO 0%, 0.5%, 1%, and 5%, all non-inoculated, and BIO 5% inoculated) of axis 2. Photosynthetic pigments and proteins strongly correlated with the inoculated control and BIO 0.2% non-inoculated. GSTs, CAT, and ETS strongly correlated with non-inoculated conditions (0%, 0.5%, 1%, and 5%). Damage (LPO and PC) and sugars correlated with inoculated conditions exposed to lower BIO microparticles.

## 4. Discussion

The surface morphology of microplastics derived from biodegradable (BIO) mulch films provides valuable insight into the degradation processes and structural alterations these materials undergo, both during use and after exposure to UV radiation. As revealed by the SEM analysis, the microplastics exhibited a range of surface textures from smooth to rugged, with some notable distinctions between weathered and non-weathered samples.

Non-weathered BIO microplastics displayed a rough texture, characterized by sharp protrusions and irregular fibre-like forms at larger size intervals. Nonetheless, the descriptions present in the literature are contradictory. While some studies, such as Convertino et al. [5] and Sun et al. [42], come in agreement with our SEM analysis, with a rough texture, other studies such as Li et al. [43] presented pristine polybutylene adipate-co-terephthalate (PBAT)-based biodegradable material as smooth. These features suggest that the significant mechanical degradation which plastic materials undergo during breakdown, such as mechanical stress, leads to ruggedness [44] as an indicator of the film’s gradual fragmentation process, where micro-cracks propagate on the surface, eventually breaking the material into smaller particles [45]. Interestingly, in our SEM images, the BIO microparticles exposed to UV weathering showed smoother surfaces, which may indicate that UV radiation promotes a degradation pathway that may lead to the scission of polymer chains, promoting surface smoothness but starting by eliminating fibre-like protrusions, since photooxidation is a surface process [46] and the highly exposed location of the protrusions and their low thickness make them a more vulnerable target to a faster degradation than the remaining parts of the particle [47]. This observation, while plausible, is not consistent with other studies that performed photodegradation using UV radiation, such as Guo et al. [48], where the presence of pores and cracks highlights that the degradation of biodegradable plastics can be distinct from case to case, possibly due to the extensive variety of different formulations of biodegradable plastics and additives to change their physical properties [49].

The ATR-FTIR analysis provided critical chemical information on the functional groups present in the biodegradable microplastics. The large peak observed between 3000 and 3600 cm^−1^, attributable to hydroxyl group vibrations, suggests that starch, or a starch-based polymer, plays a significant role in the film composition [50]. This observation aligns with the common practice of incorporating natural polymers, such as starch, into biodegradable plastics to promote biodegradation [51]. The detection of symmetric and asymmetric C–H stretching vibrations at 2847 cm^−1^ and 2917 cm^−1^, respectively, is characteristic of hydrocarbon chains, confirming the presence of aliphatic components [52]. The ATR-FTIR analysis also detected cellulose-related signals, particularly at 1020 cm^−1^, which highlights the potential presence of cellulose-based components in the biodegradable film [53]. Again, this finding is consistent with the trend of blending natural fibres or cellulose into biodegradable plastics to enhance performance [51]. More crucially, the strong C=O stretching band at 1712 cm^−1^ reveals the presence of ester bonds, particularly associated with PBAT, [54] a biodegradable polyester often used in biodegradable plastic films. This piece of data is particularly important considering that our study has a strong focus of the effects of microplastics in bacteria, most specifically PGPB. PBAT is considered a potential replacement for low-density polyethylene (LDPE) as the main component of mulch films, yet despite being a promising material, it comprises adipic acid, phthalic acid, and 1,4-butanediol as degradation products [55], compounds that present antibacterial properties [56].

After weathering, peaks at 2918 cm^−1^ and 2850 cm^−1^, related to C–H stretch, which is known to be formed after blending of PBAT with starch or cellulose [57,58] became visible in the FTIR spectre, laying evidence of a crosslinking reaction between PBAT, starch, and cellulose that the literature confirms is possible due to exposure to UV radiation [59]. Crosslinking reactions can alter the physical properties of the material as well as the degradation rate of products [60]. It should be noted that there is a considerable gap of knowledge on assessing the resulting molecules of crosslinked polymers in biodegradable plastics.

The thermogravimetric analysis of BIO microplastics, both with and without UV exposure, highlights the thermal stability and degradation profile of the material. Degradation in both weathered and untreated BIO films begins around 300 °C, with the most significant weight loss occurring between 285 °C and 400 °C, a thermal behaviour already observed in the literature [61]. This temperature range indicates the breakdown of major polymer chains, likely involving the degradation of both synthetic (e.g., PBAT) and natural (e.g., starch or cellulose) components [62,63,64].

The results of our study showed that weathering accelerates the degradation process at the higher temperature range (440 °C to 500 °C), where the material exhibits a more acute weight loss compared to the non-exposed sample. This suggests that UV weathering initiates chemical modifications in the polymer chains through Norrish Type I reactions, making the material more susceptible to thermal degradation, as was already observed by Hayes et al. [65].

In our study, the zeta potential measurement of BIO microparticles revealed a surface charge of −23.8 mV, which is confirmed by the literature [66], reporting −28.04 mV to an equivalent pH. This negative surface charge suggests that PBAT-based biodegradable microplastics, despite being considered as ecofriendly, due to their negative charge and possibly due to the presence of hydroxyl groups, may exhibit some level of hydrophilicity [67], as indicated by the ATR-FTIR analysis, which implies that the surface of these microplastics is likely to interact with cellular membranes [68], potentially inhibiting the cellular activity through altering the structure membranes, reducing molecular diffusion, and interfering with the transport of substances inside and outside cells [69], impacting organisms such as bacteria. This finding is also significant in the context of the environmental fate of such particles and interaction with soil or water particles as well, since a higher negative charge could enhance the dispersion of microplastics in aqueous environments [44] or prevent their aggregation with other negatively charged particles [70], potentially increasing their mobility and the transport of harmful chemical compounds such as metals [71]. Additionally, surface charge plays an additional role in interactions with microorganisms, as a negative zeta potential could affect biofilm formation and microbial degradation processes [72], affecting microbial mitigation mechanisms to reduce the potential toxic effect that those particles may have both on bacterial communities and individual strains. Therefore, zeta potential measurements offer insight into the potential environmental behaviour and interaction of biodegradable microplastics with their surroundings, highlighting the importance of surface chemistry in evaluating the environmental impact of biodegradable microparticles.

The widespread use of synthetic plastics as a way to create biodegradable plastics has led to an increasing accumulation of microplastics in the environment, with a composition including PBAT, a biodegradable polymer commonly used in plastic products [73]. Although considered biodegradable, PBAT microplastics may persist in ecosystems long enough to interact with microbial communities [74], raising concerns about their environmental and health impacts. To further understand the effects of biodegradable microplastics on bacteria, most specifically PGPBs, this study performed tests with five bacterial strains, *Bacillus* sp. J25, *Enterobacter ludwigii* sp. C11, *Kosakonia* sp. O21, *Rhizobium* sp. E-20-8, and *Pseudomonas* sp. S22. The inhibitory effects of BIO microparticles observed in *Bacillus*, *Enterobacter*, *Rhizobium*, and *Pseudomonas* suggest that BIO microplastics may interfere with bacterial metabolism, either through physical or chemical mechanisms, as was already seen with other microplastics [75]. One possible explanation is that the BIO microplastics themselves hinder bacterial proliferation by physically obstructing their metabolic activity, as observed by Liu et al. [1], or reducing the availability of basic nutrients [76]. However, PBAT, as a biodegradable polymer, presents additional complexity due to its ability to degrade into by-products which may further disrupt microbial activity. PBAT is known to undergo hydrolysis [77], resulting in degradation products that may accumulate in the environment [6], potentially exerting toxic or inhibitory effects on bacterial communities. While specific studies on PBAT degradation by PGPBs are lacking, the toxicity of related by-products has been documented in some bacteria. For instance, adipic acid, a key by-product of PBAT degradation, has been shown to inhibit bacterial growth at higher concentrations by generating osmotic pressure in *Escherichia coli* and *Corynebacterium glutamicum* [78]. Similar inhibitory effects could explain the suppression of *Bacillus*, *Enterobacter*, *Rhizobium*, and *Pseudomonas* in this study, although further research is required to confirm the precise mechanisms involved. In addition, the enzymatic capabilities to tolerate or degrade PBAT of these bacterial strains may have influenced their ability to grow in the presence of this contaminant. Nevertheless, there is a profound knowledge gap when it comes to the actual effects of BIO microplastics on bacteria, other than in bacterial communities, and even under that lens, the number of studies is still very insufficient to grasp the real impact that these new materials may have on bacteria, and most specifically on PGPBs. Such a gap in the study of the biological effects of BIO plastics and their degradation products, in specific strains or even genus specific reactions, prevents a fair comparison between the data of our study and the literature.

Previous research has demonstrated that some PGPBs possess enzymes such as esterases and lipases [79,80], which can degrade certain biodegradable polyesters polyhydroxyalkanoates (PHA) [81]. However, the inhibition observed in this study suggests that these strains may lack the necessary enzymatic machinery to effectively degrade PBAT or metabolize its by-products, leading to growth suppression. This raises important questions about the variability in bacterial responses to different types of biodegradable plastics and underscores the need for more detailed studies on the enzymatic pathways involved in PBAT degradation.

In contrast to the other bacterial strains in our study, *Kosakonia* showed no significant inhibition in the presence of PBAT microplastics, suggesting that this strain is able to tolerate PBAT and its degradation products. *Kosakonia* is known for its metabolic versatility and has been identified as a key player in various bioremediation processes [82], including the degradation of hydrocarbons and other xenobiotic compounds [83]; such metabolic features require enzymes capable of degrading ester bonds, which are abundant in PBAT [84]. This enzymatic activity could explain *Kosakonia*’s resilience in the presence of PBAT microplastics. Further investigation is needed to identify the specific enzymes involved and to determine if the absence of a negative interaction with BIO microplastics is due to *Kosakonia* being able to actively degrade PBAT or if it simply tolerates PBAT presence without adverse effects. Our study confirmed the ability of *Kosakonia* sp. O21 and the inability of the other bacterial strains to degrade BIO microparticles. Despite the lack of studies focusing on degradation mechanisms of PBAT by bacteria from the *Kosakonia* genus, research performed with other bacteria, such as *Pseudomonas*, *Kocuria*, *Stenotrophomonas*, *Thermobifida,* and *Actinomucor*, described the mechanisms or products involved. Proteases and lipases, the enzymes that catalyse the breaking of ester bonds in PBAT, are secreted, leading to adipic acid, terephthalic acid, benzoate, butanediol, 2-acetoin, and 3-oxoadipate when the polymer is hydrolysed. Following hydrolysis, membrane proteins intracellularly transport the smaller molecules, where they are gradually broken down. The benzoate-degradation pathway breaks down benzoate and 3-oxoadipyl-CoA, butanoate metabolism metabolizes 2-acetoin, and pyruvate and 3-oxoadipate can react with CoA, both forming acetyl-CoA, and 3-oxoadipate also forms succinyl-CoA that can be used to produce fatty acids or enter in the tricarboxylic acid cycle (TCA cycle), where they are completely metabolized to produce H_2_O and CO_2_ [85,86,87,88].

The inhibition of PGPBs by PBAT microplastics may have potential consequences for soil health and plant productivity. PGPBs, such as *Bacillus*, *Rhizobium*, *Pseudomonas*, and *Enterobacter*, play crucial roles in promoting plant growth by enhancing nutrient availability, facilitating nitrogen fixation, and increasing plant resistance to stress [89]. A reduction in the activity or abundance of these bacteria due to microplastic contamination could compromise their ability to support healthy plant growth, potentially leading to reduced crop yields and soil fertility.

Moreover, the increasing use of PBAT-based materials in agriculture, particularly in the form of biodegradable mulch films, raises concerns about the effects of BIO microplastics in soils. To elucidate how BIO microplastics influence plant growth and biochemical responses and what the role of BIO-tolerant bacterial strains in this response is, non-inoculated and *Kosakonia* sp. O21-inoculated lettuce plants were exposed to different BIO concentrations, and the plant growth and biochemical changes were evaluated.

This study highlights the profound biochemical disruptions caused by BIO microparticles, particularly in the roots of non-inoculated plants. Roots in close contact with bacteria and microparticles exhibited a higher degree of biochemical alteration than shoots. The ability of BIO microparticles, particularly the smaller ones, to penetrate the membrane and internalize within cells is likely a key factor behind this significant disruption [90,91]. Even at low concentrations (0.5%), the biochemical parameters of non-inoculated roots were notably impacted, with signs of oxidative damage. A similar result was observed by Han et al. [92] in *Brassica chinensis* exposed to PBAT microplastics, even at low concentrations (0.2%). Like in our study, Han et al. [92] also detected damage in proteins and membrane lipids. These alterations ultimately compromise the stability of the cell membranes and change cell metabolism [93,94,95], interfering in processes occurring in membranes, such as respiration and nutrient absorption [96,97] and general metabolism [98].

These disruptions directly correlate with the observed reduction in root growth, underscoring the negative impact of BIO microparticles on plant health and development. The inoculation of plants with BIO-tolerant bacteria provided partial mitigation of these effects, particularly at lower concentrations of BIO. The bacterial exopolysaccharides, whose production is increased in stress conditions [99], may play a crucial role by trapping the microparticles, thus blocking their internalization into root cells, thereby reducing the oxidative stress and biochemical damage [100]. However, this protective effect failed to fully counteract the effects at higher BIO concentrations (1% and 5%). At these levels, the accumulation of ROS that the increased activity of antioxidant enzymes was not able to irradicate conducted to cell damage, though it was less severe compared to non-inoculated plants.

In the shoots, our study also revealed biochemical alterations, though to a lesser degree than in roots. Photosynthetic pigments were decreased, electron transport system activity was increased, and antioxidant enzymes, particularly superoxide dismutase (SOD) and glutathione-S-transferases (GSTs), also increased. These responses were able to control cell damage, but shoot growth was highly impacted, since the decrease in photosynthetic pigments certainly decreased the production of photosynthates, and the higher energy expenditure (higher ETS activity) to combat oxidative stress left fewer resources available for growth. Sun et al. [101] for *Zea mays* and Wang et al. [102] for *Lactuca sativa* also reported decreases in photosynthetic pigments when plants were exposed to BIO microplastics.

Appenroth et al. [103] explained that the increase in cellular respiration will increase the production of energy used to counteract the abiotic stress generated by the microparticles. In our study, non-inoculated plants increased ETS activity, dispensing energy to fight the cellular changes, like oxidative stress, induced by BIO particles. However, lower ETS activity was observed in inoculated plants, which may be due to a more efficient use of energy or to the protective action of bacteria towards the toxicity effects induced by BIO.

The findings of this study align with other reports that BIO microparticles, potentially containing PBAT derivatives, cause severe biochemical disruptions in plants [6,74]. This correlation underscores the broader environmental risks associated with biodegradable microplastics and suggests that current formulations, even with bacterial inoculants, may not sufficiently protect plants from these toxic effects.

Thus, while the inoculation of plants with BIO-tolerant bacteria offers some potential for mitigating the effects of biodegradable microplastics, particularly at lower concentrations, it does not emerge as a robust solution for higher concentrations of BIO. These findings suggest a need for the development of more effective inoculants or alternative strategies to protect plants from the toxic effects of PBAT-derived microparticles. This work emphasizes the importance of understanding the biochemical impacts of biodegradable microplastics in soil and highlights the necessity of sustainable approaches to ensure agricultural productivity in microplastic-contaminated soils.

## 5. Conclusions

The findings of this study identify significant gaps in understanding the interactions between biodegradable microplastics derived from BIO mulch films and plant growth-promoting bacteria (PGPB), which could have considerable impacts on soil health, microbial dynamics, and sustainable agriculture. Through a detailed examination of the interaction between biodegradable microplastics and five bacterial strains, *Bacillus*, *Enterobacter*, *Kosakonia*, *Rhizobium*, and *Pseudomonas*, this research highlights varying and strain-specific responses to BIO microplastic exposure. This study also highlights the dual effects of UV weathering on the degradation of microplastics, which changed the particle’s chemical and physical characteristics and affected microbial tolerance to BIO microplastics. One important finding from this study is that most bacterial strains are negatively affected by BIO microplastics. This inhibition may be caused by surface charge changes and chemical byproducts of PBAT decomposition, which could disrupt bacterial metabolism. However, *Kosakonia* sp. O21 demonstrated notable tolerance to these microplastics, suggesting that certain bacterial strains may possess mechanisms to survive or degrade BIO microplastics. This study also explored the effects of these microplastics on plant biochemistry, showing oxidative stress, disruption of the photosynthetic processes, and reduced growth in non-inoculated plants. Bacterial inoculation offered partial mitigation, especially at lower concentrations of microplastics. These findings have important implications for sustainable agricultural practices, as biodegradable microplastics may not fully eliminate the risks posed to soil ecosystems. Although BIO materials are designed to break down more readily than traditional plastics, their degradation products and interactions with microbial communities and plants can still present significant ecological risks. Developing microbial inoculants that can either degrade these microplastics or protect plants from their harmful effects will be crucial for mitigating the negative consequences on crop productivity and soil health. Moreover, further research is essential to extend these findings beyond controlled conditions, exploring how these interactions unfold in field conditions where factors like soil composition, climate, and biodiversity may influence outcomes. Long-term studies will be required to assess the persistence of biodegradable microplastics, their temporal effects on soil microbial communities, and the potential for bioinoculants to provide lasting protection to crops. This study adds to our understanding of the effects that biodegradable microplastics may pose to agroecosystems, in particular to microbial communities, and specifically for those microorganisms that promote plant growth, opening new paths for biotechnological solutions to allow the use of biodegradable mulch films without putting crop productivity in jeopardy. Using microbial-based techniques will be essential to supporting sustainable farming, safeguarding soil health, and lowering the environmental impact of agriculture in the context of growing microplastic pollution.

## Data Availability

The data presented in this study are available on request from the corresponding author.

## References

[1] Li Y., Chen Y., Li P., Huang H., Xue K., Cai S., Liao X., Jin S., Zheng D. (2024). Microplastics in soil affect the growth and physiological characteristics of Chinese fir and *Phoebe bournei* seedlings. Environ. Pollut..

[2] Menossi M., Cisneros M., Alvarez V.A., Casalongué C. (2021). Current and emerging biodegradable mulch films based on polysaccharide bio-composites. A review. Agron. Sustain. Dev..

[3] Song Z., Zhao L., Bi J., Tang Q., Wang G., Li Y. (2024). Classification of Degradable Mulch Films and Their Promotional Effects and Limitations on Agricultural Production. Agriculture.

[4] Moshood T.D., Nawanir G., Mahmud F., Mohamad F., Ahmad M.H., AbdulGhani A. (2022). Sustainability of biodegradable plastics: New problem or solution to solve the global plastic pollution?. Curr. Res. Green Sustain. Chem..

[5] Convertino F., Carroccio S.C., Cocca M.C., Dattilo S., Dell’Acqua A.C., Gargiulo L., Nizzetto L., Riccobene P.M., Schettini E., Vox G. (2024). The fate of post-use biodegradable PBAT-based mulch films buried in agricultural soil. Sci. Total Environ..

[6] Martínez A., Perez-Sanchez E., Caballero A., Ramírez R., Quevedo E., Salvador-García D. (2024). PBAT is biodegradable but what about the toxicity of its biodegradation products?. J. Mol. Model..

[7] (2016). Plastics—Methods of Exposure to Laboratory Light Sources, Part 3: Fluorescent UV Lamps.

[8] Pinto S.C., Marques P.A.A.P., Vicente R., Godinho L., Duarte I. (2020). Hybrid Structures Made of Polyurethane/Graphene Nanocomposite Foams Embedded within Aluminum Open-Cell Foam. Metals.

[9] Gonçalves G., Domingues E.M., Ferreira N., Ranawadia K., Henriques B., Bessa A., Tavares D., Martins N., Pereira E., Marques P.A. (2024). Enhanced Hg(II) removal using Thiourea-Functionalized graphene Oxide: Lab to pilot scale evaluation. Sep. Purif. Technol..

[10] Trindade N. (2022). Use of *Bacillus* sp. in Cleaning Products. Master’s Thesis.

[11] Lopes T., Cardoso P., Matos D., Rocha R., Pires A., Marques P., Figueira E. (2022). Graphene oxide influence in soil bacteria is dose dependent and changes at osmotic stress: Growth variation, oxidative damage, antioxidant response, and plant growth promotion traits of a *Rhizobium* strain. Nanotoxicology.

[12] Wróbel M., Szymańska S., Kowalkowski T., Hrynkiewicz K. (2022). Selection of microorganisms capable of polyethylene (PE) and polypropylene (PP) degradation. Microbiol. Res..

[13] Rocha R., Lopes T., Fidalgo C., Alves A., Cardoso P., Figueira E. (2022). Bacteria Associated with the Roots of Common Bean (*Phaseolus vulgaris* L.) at Different Development Stages: Diversity and Plant Growth Promotion. Microorganisms.

[14] Figueira E. (2000). Aspectos da Tolerância Salina em *Pisum sativum* L.: Influência da Nutrição Azotada. Ph.D. Thesis.

[15] Cardoso P., Freitas R., Figueira E. (2014). Salt tolerance of rhizobial populations from contrasting environmental conditions: Understanding the implications of climate change. Ecotoxicology.

[16] Lopes T., Cruz C., Cardoso P., Pinto R., Marques PA A.P., Figueira E. (2021). A Multifactorial Approach to Untangle Graphene Oxide (GO) Nanosheets Effects on Plants: Plant Growth-Promoting Bacteria Inoculation, Bacterial Survival, and Drought. Nanomaterials.

[17] Robinson H.W., Hogden C.G. (1940). The biuret reaction in the determination of serum proteins. J. Biol. Chem..

[18] Sekar S., Mahadevan S., Kumar SS D., Mandal A.B. (2010). Thermokinetic responses of the metabolic activity of *Staphylococcus lentus* cultivated in a glucose limited mineral salt medium. J. Therm. Anal. Calorim..

[19] Ilahi WF F., Ahmad D., Husain M.C. (2017). Effects of root zone cooling on butterhead lettuce grown in tropical conditions in a coir-perlite mixture. Hortic. Environ. Biotechnol..

[20] Rouphael Y., Cardarelli M., Bassal A., Leonardi C., Giuffrida F., Colla G. (2012). Vegetable quality as affected by genetic*Agronomic and environmental factors. J. Food Agric. Environ..

[21] Malinconico M. (2017). Soil Degradable Bioplastics for a Sustainable Modern Agriculture. Green Chemistry and Sustainable Technology.

[22] Murashige T., Skoog F. (1962). A Revised Medium for Rapid Growth and Bio Assays with Tobacco Tissue Cultures. Physiol. Plant..

[23] Wang W., Ge J., Yu X., Li H. (2019). Environmental fate and impacts of microplastics in soil ecosystems: Progress and perspective. Sci. Total Environ..

[24] Wellburn A.R., Lichtenthaler H. (1984). Formulae and Program to Determine Total Carotenoids and Chlorophylls A and B of Leaf Extracts in Different Solvents. Advances in Photosynthesis Research: Proceedings of the VIth International Congress on Photosynthesis, Brussels, Belgium, August 1–6, 1983 Volume 2.

[25] Cardoso P., Pinto R., Lopes T., Figueira E. (2024). How Bacteria Cope with Oxidative Stress Induced by Cadmium: Volatile Communication Is Differentially Perceived Among Strains. Antioxidants.

[26] King F.D., Packard T.T. (1975). Respiration and the activity of the respiratory electron transport system in marine zooplankton. Limnol. Oceanogr..

[27] Mesquita C.S., Oliveira R., Bento F., Geraldo D., Rodrigues J.V., Marcos J.C. (2014). Simplified 2,4-dinitrophenylhydrazine spectrophotometric assay for quantification of carbonyls in oxidized proteins. Anal. Biochem..

[28] Beauchamp C., Fridovich I. (1971). Superoxide dismutase: Improved assays and an assay applicable to acrylamide gels. Anal. Biochem..

[29] Johansson L.H., Borg L.H. (1988). A spectrophotometric method for determination of catalase activity in small tissue samples. Anal. Biochem..

[30] DuBois M., Gilles K.A., Hamilton J.K., Rebers P.A., Smith F. (1956). Colorimetric Method for Determination of Sugars and Related Substances. Anal. Chem..

[31] Buege J.A., Aust S.D. (1978). Microsomal lipid peroxidation. Methods Enzymol..

[32] Habig W.H., Pabst M.J., Jakoby W.B. (1974). Glutathione S-Transferases. J. Biol. Chem..

[33] Garces V., García-Quintero A., Lerma T.A., Palencia M., Combatt E.M., Arrieta Á.A. (2021). Characterization of Cassava Starch and Its Structural Changes Resulting of Thermal Stress by Functionally-Enhanced Derivative Spectroscopy (FEDS). Polysaccharides.

[34] De Oliveira AC S., Santos T.A., Ugucioni J.C., Da Rocha R.A., Borges S.V. (2021). Effect of glycerol on electrical conducting of chitosan/polyaniline blends. J. Appl. Polym. Sci..

[35] Laorenza Y., Harnkarnsujarit N. (2024). Surface adhesion and physical properties of modified TPS and PBAT multilayer film. Food Packag. Shelf Life.

[36] Siew Z.Z., Chan EW C., Wong C.W. (2023). Enhancing the Tearability and Barrier Properties of Cellulose Acetate Bioplastic Film with Polyethylene Glycol 1450 as an LDPE Replacement for Food Packaging. Food Bioprocess Technol..

[37] Nunes F.C., Ribeiro K.C., Martini F.A., Barrioni B.R., Santos J.P.F., Carvalho B.M. (2021). PBAT/PLA/cellulose nanocrystals biocomposites compatibilized with polyethylene grafted maleic anhydride (PE-g-MA). J. Appl. Polym. Sci..

[38] Syazwani N.S., Efzan M.E., Kok C., Nurhidayatullaili M. (2021). Analysis on extracted jute cellulose nanofibers by Fourier transform infrared and X-Ray diffraction. J. Build. Eng..

[39] Bonilla J., Paiano R.B., Lourenço R.V., Bittante AM Q., Sobral P.J. (2020). Biodegradability in aquatic system of thin materials based on chitosan, PBAT and HDPE polymers: Respirometric and physical-chemical analysis. Int. J. Biol. Macromol..

[40] Ko Y., Yang Y., Kim D., Lee Y.H., Ghatge S., Hur H. (2024). Fungal biodegradation of poly(butylene adipate-co-terephthalate)-polylactic acid-thermoplastic starch based commercial bio-plastic film at ambient conditions. Chemosphere.

[41] Skvorčinskienė R., Kiminaitė I., Vorotinskienė L., Jančauskas A., Paulauskas R. (2023). Complex study of bioplastics: Degradation in soil and characterization by FTIR-ATR and FTIR-TGA methods. Energy.

[42] Sun H., Jiao R., Wang D. (2020). The difference of aggregation mechanism between microplastics and nanoplastics: Role of Brownian motion and structural layer force. Environ. Pollut..

[43] Li C., Cui Q., Li Y., Zhang K., Lu X., Zhang Y. (2022). Effect of LDPE and biodegradable PBAT primary microplastics on bacterial community after four months of soil incubation. J. Hazard. Mater..

[44] Sun J., Zheng H., Xiang H., Fan J., Jiang H. (2022). The surface degradation and release of microplastics from plastic films studied by UV radiation and mechanical abrasion. Sci. Total Environ..

[45] Zaidi Z., Mawad D., Crosky A. (2019). Soil Biodegradation of Unidirectional Polyhydroxybutyrate-Co-Valerate (PHBV) Biocomposites Toughened with Polybutylene-Adipate-Co-Terephthalate (PBAT) and Epoxidized Natural Rubber (ENR). Front. Mater..

[46] Sun J., Wang X., Zheng H., Xiang H., Jiang X., Fan J. (2023). Characterization of the degradation products of biodegradable and traditional plastics on UV irradiation and mechanical abrasion. Sci. Total Environ..

[47] Tong H., Zhong X., Duan Z., Yi X., Cheng F., Xu W., Yang X. (2022). Micro- and nanoplastics released from biodegradable and conventional plastics during degradation: Formation, aging factors, and toxicity. Sci. Total Environ..

[48] Guo C., Wang L., Lang D., Qian Q., Wang W., Wu R., Wang J. (2022). UV and chemical aging alter the adsorption behavior of microplastics for tetracycline. Environ. Pollut..

[49] Kim M.S., Chang H., Zheng L., Yan Q., Pfleger B.F., Klier J., Nelson K., Majumder E.L., Huber G.W. (2023). A Review of Biodegradable Plastics: Chemistry, Applications, Properties, and Future Research Needs. Chem. Rev..

[50] Demirgöz D., Elvira C., Mano J.F., Cunha A.M., Piskin E., Reis R.L. (2000). Chemical modification of starch based biodegradable polymeric blends: Effects on water uptake, degradation behaviour and mechanical properties. Polym. Degrad. Stab..

[51] Gunawardene OH P., Gunathilake C., Amaraweera S.M., Fernando NM L., Wanninayaka D.B., Manamperi A., Kulatunga A.K., Rajapaksha S.M., Dassanayake R.S., Fernando C.A.N. (2021). Compatibilization of Starch/Synthetic Biodegradable Polymer Blends for Packaging Applications: A Review. J. Compos. Sci..

[52] Campanale C., Savino I., Massarelli C., Uricchio V.F. (2023). Fourier Transform Infrared Spectroscopy to Assess the Degree of Alteration of Artificially Aged and Environmentally Weathered Microplastics. Polymers.

[53] Liu M., Tong S., Tong Z., Guan Y., Sun Y. (2023). A strong, biodegradable and transparent cellulose-based bioplastic stemmed from waste paper. J. Appl. Polym. Sci..

[54] Nomadolo N., Dada O.E., Swanepoel A., Mokhena T., Muniyasamy S. (2022). A Comparative Study on the Aerobic Biodegradation of the Biopolymer Blends of Poly(butylene succinate), Poly(butylene adipate terephthalate) and Poly(lactic acid). Polymers.

[55] Liu L., Zou G., Zuo Q., Li S., Bao Z., Jin T., Liu D., Du L. (2022). It is still too early to promote biodegradable mulch film on a large scale: A bibliometric analysis. Environ. Technol. Innov..

[56] Zhao Z., Balu R., Gangadoo S., Duta N.K., Choudhury N.R. (2024). Poly(butylene adipate-co-terephthalate)/Polylactic Acid/Tetrapod-Zinc Oxide Whisker Composite Films with Antibacterial Properties. Polymers.

[57] Giri J., Lach R., Le H.H., Grellmann W., Saiter J., Henning S., Radusch H., Adhikari R. (2020). Structural, thermal and mechanical properties of composites of poly(butylene adipate-co-terephthalate) with wheat straw microcrystalline cellulose. Polym. Bull..

[58] Nobrega M.M., Olivato J.B., Müller CM O., Yamashita F. (2012). Biodegradable starch-based films containing saturated fatty acids: Thermal, infrared and raman spectroscopic characterization. Polímeros.

[59] Bian X., Fan S., Xia G., Xin J.H., Jiang S. (2024). Effect of UV-induced crosslink network structure on the properties of polylactic acid/polybutylene adipate terephthalate blend. J. Polym. Res..

[60] Zhang Z., Yu Z., Zhang X., Shan T., Li L., Deng T., Zhang Z. (2023). Improving the Foaming Behavior of PBAT by Graft Modification: Mechanisms, Characteristics, and Degradation. J. Polym. Environ..

[61] Pokhrel S., Sigdel A., Lach R., Slouf M., Sirc J., Katiyar V., Bhattarai D.R., Adhikari R. (2021). Starch-based biodegradable film with poly(butylene adipate-co-terephthalate): Preparation, morphology, thermal and biodegradation properties. J. Macromol. Sci. Part A.

[62] Chen W., Qi C., Li Y., Tao H. (2020). The degradation investigation of biodegradable PLA/PBAT blend: Thermal stability, mechanical properties and PALS analysis. Radiat. Phys. Chem..

[63] Liu X., Yu L., Liu H., Chen L., Li L. (2007). In situ thermal decomposition of starch with constant moisture in a sealed system. Polym. Degrad. Stab..

[64] Yang H., Yan R., Chen H., Lee D.H., Zheng C. (2007). Characteristics of hemicellulose, cellulose and lignin pyrolysis. Fuel.

[65] Hayes D.G., Wadsworth L.C., Sintim H.Y., Flury M., English M., Schaeffer S., Saxton A.M. (2017). Effect of diverse weathering conditions on the physicochemical properties of biodegradable plastic mulches. Polym. Test..

[66] Maciel C.C., De Barros A., Mazali I.O., Ferreira M. (2023). Flexible biodegradable electrochemical sensor of PBAT and CNDs composite for the detection of emerging pollutants. J. Electroanal. Chem..

[67] Bao Z., Chen Z., Lu S., Wang G., Qi Z., Cai Z. (2021). Effects of hydroxyl group content on adsorption and desorption of anthracene and anthrol by polyvinyl chloride microplastics. Sci. Total Environ..

[68] Qian J., He X., Wang P., Xu B., Li K., Lu B., Jin W., Tang S. (2021). Effects of polystyrene nanoplastics on extracellular polymeric substance composition of activated sludge: The role of surface functional groups. Environ. Pollut..

[69] Rossi G., Barnoud J., Monticelli L. (2013). Polystyrene Nanoparticles Perturb Lipid Membranes. J. Phys. Chem. Lett..

[70] Yang X., An C., Feng Q., Boufadel M., Ji W. (2022). Aggregation of microplastics and clay particles in the nearshore environment: Characteristics, influencing factors, and implications. Water Res..

[71] Tang S., Lin L., Wang X., Yu A., Sun X. (2020). Interfacial interactions between collected nylon microplastics and three divalent metal ions (Cu(II), Ni(II), Zn(II)) in aqueous solutions. J. Hazard. Mater..

[72] Qin Y., Tu Y., Chen C., Wang F., Yang Y., Hu Y. (2024). Biofilms on microplastic surfaces and their effect on pollutant adsorption in the aquatic environment. J. Mater. Cycles Waste Manag..

[73] Jian J., Xiangbin Z., Xianbo H. (2020). An overview on synthesis, properties and applications of poly(butylene-adipate-co-terephthalate)–PBAT. Adv. Ind. Eng. Polym. Res..

[74] Liu J., Wang P., Wang Y., Zhang Y., Xu T., Zhang Y., Xi J., Hou L., Li L., Zhang Z. (2022). Negative effects of poly(butylene adipate-co-terephthalate) microplastics on *Arabidopsis* and its root-associated microbiome. J. Hazard. Mater..

[75] Ning Q., Wang D., An J., Ding Q., Huang Z., Zou Y., Wu F., You J. (2021). Combined effects of nanosized polystyrene and erythromycin on bacterial growth and resistance mutations in *Escherichia coli*. J. Hazard. Mater..

[76] Li X., Zheng G., Li Z., Fu P. (2023). Formulation, performance and environmental/agricultural benefit analysis of biomass-based biodegradable mulch films: A review. Eur. Polym. J..

[77] Kijchavengkul T., Auras R., Rubino M., Selke S., Ngouajio M., Fernandez R.T. (2010). Biodegradation and hydrolysis rate of aliphatic aromatic polyester. Polym. Degrad. Stab..

[78] Karlsson E., Mapelli V., Olsson L. (2017). Adipic acid tolerance screening for potential adipic acid production hosts. Microb. Cell Factories.

[79] Bagheri N., Ahmadzadeh M., Mariotte P., Jouzani G.S. (2022). Behavior and interactions of the plant growth-promoting bacteria *Azospirillum oryzae* NBT506 and *Bacillus velezensis* UTB96 in a co-culture system. World J. Microbiol. Biotechnol..

[80] Singh R., Pandey K.D., Singh M., Singh S.K., Hashem A., Al-Arjani A.F., Abd_Allah E.F., Singh P.K., Kumar A. (2022). Isolation and Characterization of Endophytes Bacterial Strains of *Momordica charantia* L. and Their Possible Approach in Stress Management. Microorganisms.

[81] Wu P., Li Z., Gao J., Zhao Y., Wang H., Qin H., Gu Q., Wei R., Liu W., Han X. (2023). Characterization of a PBAT Degradation Carboxylesterase from *Thermobacillus composti* KWC4. Catalysts.

[82] Dash D.M., Osborne W.J. (2020). Rapid biodegradation and biofilm-mediated bioremoval of organophosphorus pesticides using an indigenous *Kosakonia oryzae* strain-VITPSCQ3 in a Vertical-flow Packed Bed Biofilm Bioreactor. Ecotoxicol. Environ. Saf..

[83] Choudhury S.P., Panda S., Haq I., Kalamdhad A.S. (2022). Microbial pretreatment using *Kosakonia oryziphila* IH3 to enhance biogas production and hydrocarbon depletion from petroleum refinery sludge. Renew. Energy.

[84] Leelaphiwat P., Pechprankan C., Siripho P., Bumbudsanpharoke N., Harnkarnsujarit N. (2021). Effects of nisin and EDTA on morphology and properties of thermoplastic starch and PBAT biodegradable films for meat packaging. Food Chem..

[85] Jia H., Zhang M., Weng Y., Li C. (2020). Degradation of polylactic acid/polybutylene adipate-co-terephthalate by coculture of *Pseudomonas mendocina* and *Actinomucor elegans*. J. Hazard. Mater..

[86] Jia H., Zhang M., Weng Y., Zhao Y., Li C., Kanwal A. (2020). Degradation of poly(butylene adipate-co-terephthalate) by *Stenotrophomonas* sp. YCJ1 isolated from farmland soil. J. Environ. Sci..

[87] Jia X., Zhao K., Zhao J., Lin C., Zhang H., Chen L., Chen J., Fang Y. (2022). Degradation of poly(butylene adipate-co-terephthalate) films by *Thermobifida fusca* FXJ-1 isolated from compost. J. Hazard. Mater..

[88] Qiu Y., Wang P., Zhang L., Li C., Lu J., Ren L. (2024). Enhancing biodegradation efficiency of PLA/PBAT-ST20 bioplastic using thermophilic bacteria co-culture system: New insight from structural characterization, enzyme activity, and metabolic pathways. J. Hazard. Mater..

[89] Cruz C., Cardoso P., Santos J., Matos D., Sá C., Figueira E. (2023). Application of Plant Growth-Promoting Bacteria from Cape Verde to Increase Maize Tolerance to Salinity. Antioxidants.

[90] Mateos-Cárdenas A., Van Pelt F.N., O’Halloran J., Jansen M.A. (2021). Adsorption, uptake and toxicity of micro- and nanoplastics: Effects on terrestrial plants and aquatic macrophytes. Environ. Pollut..

[91] Yu Z., Xu X., Guo L., Jin R., Lu Y. (2023). Uptake and transport of micro/nanoplastics in terrestrial plants: Detection, mechanisms, and influencing factors. Sci. Total Environ..

[92] Han Y., Teng Y., Wang X., Wen D., Gao P., Yan D., Yang N. (2023). Biodegradable PBAT microplastics adversely affect pakchoi (*Brassica chinensis* L.) growth and the rhizosphere ecology: Focusing on rhizosphere microbial community composition, element metabolic potential, and root exudates. Sci. Total Environ..

[93] Chakraborty N., Mitra R., Dasgupta D., Ganguly R., Acharya K., Minkina T., Popova V., Churyukina E., Keswani C. (2023). Unraveling lipid peroxidation-mediated regulation of redox homeostasis for sustaining plant health. Plant Physiol. Biochem..

[94] Sadžak A., Mravljak J., Maltar-Strmečki N., Arsov Z., Baranović G., Erceg I., Kriechbaum M., Strasser V., Přibyl J., Šegota S. (2020). The Structural Integrity of the Model Lipid Membrane during Induced Lipid Peroxidation: The Role of Flavonols in the Inhibition of Lipid Peroxidation. Antioxidants.

[95] Sun M., Peng F., Xiao Y., Yu W., Zhang Y., Gao H. (2019). Exogenous phosphatidylcholine treatment alleviates drought stress and maintains the integrity of root cell membranes in peach. Sci. Hortic..

[96] Roy T., Dey T.K., Jamal M. (2022). Microplastic/nanoplastic toxicity in plants: An imminent concern. Environ. Monit. Assess..

[97] Yu Z., Xu X., Guo L., Yuzuak S., Lu Y. (2024). Physiological and biochemical effects of polystyrene micro/nano plastics on *Arabidopsis thaliana*. J. Hazard. Mater..

[98] Yang C., Gao X. (2022). Impact of microplastics from polyethylene and biodegradable mulch films on rice (*Oryza sativa* L.). Sci. Total Environ..

[99] Jeong S., Kim T., Choi B., Kim Y., Kim E. (2021). Invasive *Lactuca serriola* seeds contain endophytic bacteria that contribute to drought tolerance. Sci. Rep..

[100] Pathan S.I., Arfaioli P., Bardelli T., Ceccherini M.T., Nannipieri P., Pietramellara G. (2020). Soil Pollution from Micro- and Nanoplastic Debris: A Hidden and Unknown Biohazard. Sustainability.

[101] Sun H., Shi Y., Zhao P., Long G., Li C., Wang J., Qiu D., Lu C., Ding Y., Liu L. (2023). Effects of polyethylene and biodegradable microplastics on photosynthesis, antioxidant defense systems, and arsenic accumulation in maize (*Zea mays* L.) seedlings grown in arsenic-contaminated soils. Sci. Total Environ..

[102] Wang W., Xie Y., Li H., Dong H., Li B., Guo Y., Wang Y., Guo X., Yin T., Liu X. (2023). Responses of lettuce (*Lactuca sativa* L.) growth and soil properties to conventional non-biodegradable and new biodegradable microplastics. Environ. Pollut..

[103] Rozman U., Kalčíková G. (2022). The Response of Duckweed *Lemna minor* to Microplastics and Its Potential Use as a Bioindicator of Microplastic Pollution. Plants.

